# Psychometric properties of the novel patient-reported Functional Impacts of Narcolepsy Instrument (FINI) using data from phase 3 clinical trials in narcolepsy type 1

**DOI:** 10.3389/fneur.2026.1884901

**Published:** 2026-10-15

**Authors:** Heather Romero, Yulia Savva, Stephen Crawford, Amy Howerter, Shruti Dave, Helen Doll

**Affiliations:** 1 Takeda Development Center Americas, Inc., Cambridge, MA, United States; 2 Clinical Outcomes Solutions, Tucson, AZ, United States; 3 Clinical Outcomes Solutions, Folkestone, United Kingdom

**Keywords:** FINI, Functional Impacts of Narcolepsy Instrument, narcolepsy, narcolepsy type 1, NT1, patient-reported outcomes, psychometric

## Abstract

**Background:**

The Functional Impacts of Narcolepsy Instrument (FINI) is a 28-item patient-reported outcome measure developed to evaluate key functional impacts in people with narcolepsy type 1 (NT1) and 2 (NT2) across 6 domains: Tiredness, Cognitive Functioning, Cataplexy, Social Activities, Everyday Activities, Everyday Responsibilities. This study aimed to assess the FINI's psychometric properties and estimate meaningful within-patient change (MWPC) thresholds in NT1 using pooled data from 2 phase 3 clinical trials evaluating oveporexton in NT1 (*N* = 167, NCT06470828; *N* = 105, NCT06505031).

**Methodology:**

FINI scale structure, known-groups validity, convergent/divergent validity, internal consistency reliability, test-retest reliability, and responsiveness were evaluated. MWPC thresholds were estimated using anchor- and distribution-based approaches.

**Results:**

Confirmatory factor analysis supported the FINI 6-domain scale structure. The domains demonstrated good known-groups validity, especially for comparisons between severe and moderate reference measure groups (most *P*-values < 0.05, standardized effect sizes: 0.3–0.9). Convergent/divergent validity was supported by agreement between observed and expected correlation coefficients between FINI domains and reference measures. Most domains demonstrated good internal consistency (Cronbach's alpha: 0.63–0.90; ≥0.78 for all except Everyday Activities). Excellent test-retest reliability was observed for all domains in a subpopulation of participants with a stable condition based on self-reported Patient Global Impression of Severity scores (ICC ≥0.70). Good responsiveness was demonstrated for all FINI domains, supported by correlations between domain and continuous reference measure change scores >0.35, and with greater improvements in domain scores associated with greater categorical improvements in reference measures (all *P*-values <0.05, average between-group standardized response mean across domains: 0.69). All prespecified anchors exceeded the *a priori* threshold correlation with FINI domains (≥0.30) and were used to derive MWPC thresholds (ranges): Tiredness and Everyday Activities, −35 (−30 to −40); Cognitive Functioning and Everyday Responsibilities, −30 (−25 to −35); Social Activities, −25 (−20 to −30); and Cataplexy, −20 (−15 to −25).

**Conclusions:**

The FINI is a robust and valid measure assessing meaningful and important key functional impacts for people with narcolepsy. These findings support use of the instrument in clinical trials to evaluate treatment benefit in NT1. Given the FINI's sensitivity to change, it can potentially facilitate treatment decisions and track longitudinal outcomes in clinical practice.

## 1 Introduction

Narcolepsy is a rare, chronic, neurological central disorder of hypersomnolence characterized by symptoms affecting sleep-wake regulation, most notably excessive daytime sleepiness (EDS), disturbed nighttime sleep, hypnagogic/hypnopompic hallucinations, and sleep paralysis (1–3). The pentad symptoms include four symptoms of sleep-wake dysregulation as well as cataplexy (characterized by sudden brief episodes of muscle weakness). Narcolepsy is classified into two main types: type 1 (NT1) and type 2 (NT2) (1, 3), with NT1 distinguished from NT2 by the presence of cataplexy. Narcolepsy symptoms frequently impact patients' health-related quality of life (HRQoL) and functionally impact work/school performance, cognition, and other activities of daily living (2, 4–6). Despite this substantial burden (7, 8), there are limited patient-reported outcome (PRO) measures specifically available to evaluate functional outcomes for people with narcolepsy in clinical trials or clinical practice.

Currently utilized measures for assessing narcolepsy symptoms include the Epworth Sleepiness Scale (ESS) (9), Stanford Sleepiness Scale (10), Insomnia Severity Index (11), Pittsburgh Sleep Quality Index (12), Functional Outcomes of Sleep Questionnaire (FOSQ) (13), and the Karolinska Sleepiness Scale (14), with the ESS being the most frequently used (15, 16). These instruments focus on measuring EDS symptoms, but do not capture the full experience of people living with narcolepsy. The Narcolepsy Severity Scale (NSS) and NSS for Clinical Trials (NSS-CT) measure the pentad of narcolepsy symptoms and impacts and are used for classifying severity of symptoms (17, 18). Additionally, the Patient-reported Outcome Measure for Central Disorders of Hypersomnolence (PROM-CDH) goes beyond measurement of pentad symptoms and is the first tool to measure HRQoL outcomes in people with NT1, NT2, and idiopathic hypersomnia (IH) (19). The PROM-CDH measures the full impact of CDH on quality of life and covers 5 domains (Outlook on Life; Energy, Attention, and Activities; Coping with CDH; Physical Wellbeing; and Impact of Cataplexy), as well as 5 stand-alone items (daytime sleepiness, nap[s], driving a car, public transport, and sexual activity) (19).

The Functional Impacts of Narcolepsy Instrument (FINI) was developed to address the need for a narcolepsy-specific measure that assesses functional outcomes, thus adding to the body of existing PROs (8). The measure was developed with input from patients with narcolepsy, clinical experts in narcolepsy management, and clinical outcome scientists, following patient-focused drug development guidance established by the United States (US) Food and Drug Administration for the development of clinical outcomes assessments (COAs) (20). Qualitative interviews among people with narcolepsy conducted as part of early development of the FINI (8) indicate that the functional impacts most frequently reported by people with narcolepsy are related to EDS, cataplexy, and cognitive difficulties. For example, these symptoms interfere with their ability to function throughout the day (e.g., walking, driving, cooking, and household chores), resulting in disruptions to work or school (e.g., stopped/decreased attendance at work or school, change in work or career plans), and limitations in social and leisure activities (8). The FINI measures functional impacts of narcolepsy across the 6 domains patients indicated were most frequently impacted by narcolepsy, namely Tiredness, Cognitive Functioning, Cataplexy (NT1 only), Social Activities, Everyday Activities, and Everyday Responsibilities.

During development of the FINI, NT1 and NT2 were selected as the target populations based on historical and clinical considerations. However, given differences in the patient experience between NT1 and NT2, including diagnostic instability of NT2 (21, 22), we leveraged literature from both NT1 and NT2 but conducted psychometric research separately. In addition, due to the unique features of IH related to clinical characteristics and diagnostic stability (22), FINI development in this population is currently being investigated separately. Historically, NT1 and NT2 have often been studied together in clinical trials and were jointly represented in the FDA's 2014 Voice of the Patient report on narcolepsy (23). To inform instrument development, separate concept elicitation interviews were conducted with participants with NT1 and NT2 (8). Saturation analyses conducted separately for each population demonstrated substantial overlap in the symptoms and impacts reported by the two populations, with the exception of cataplexy, which is specific to NT1. These findings supported the co-development of a single instrument for use in both populations. In alignment with the FDA's preference to provide indication-specific evidence, the current report outlines psychometric evidence for the FINI specific to NT1. Evidence for the FINI-NT2 and FINI-IH is not included in the current report.

During development, a previous version of the FINI demonstrated strong psychometric properties in a population of people with NT1 from an observational study and a Phase 2 study with similar clinical and age characteristics to those of NT1 clinical trial populations (24). Here, we describe the psychometric evaluation of the final FINI v1.0 and estimate meaningful within-patient change (MWPC) thresholds in people with NT1 using data from 2 phase 3 clinical trials.

## 2 Methods

### 2.1 Study design and assessments

The development of the FINI and the scoring methods (based on qualitative patient interviews, exploratory/confirmatory factor analyses [CFA], and Rasch analysis) have been reported previously (8). Briefly, FINI item generation was derived from concept elicitation interviews and refined with cognitive debriefing interviews with study participants. The FINI scale structure was determined using two independent datasets: a cross-sectional, US observational study enrolling participants with NT1; and a global phase 2 study enrolling participants with NT1 and NT2 (TAK-994-1501, NCT04096560 (25)). The final FINI (the FINI v1.0) comprises 28 items across 6 domains, as supported by CFA (Table 1). All items use a 5-point response scale (scored 0–4) over a 7-day recall, with higher scores indicating greater symptom severity or frequency. Administration time takes approximately 5 min. An average score is calculated for each domain, which is standardized to a 0–100 scale, where 0 indicates the best health and 100 the worst health. The English language version of the FINI was translated and linguistically validated into the local language(s) of each country according to the International Society for Pharmacoeconomics and Outcomes Research (ISPOR) *Principles of Good Practice for the Translation and Cultural Adaptation Process for Patient-Reported Outcomes (PRO) Measures* (26).

**Table 1:** Summary of the FINI^*^.

Domains	Item concepts	Response options
Tiredness	Feel fatigued	• Not at all (0) • A little bit (1) • Somewhat (2) • Quite a bit (3) • Very much (4)
	Tired	
	Trouble starting things	
	Trouble finishing things	
	Frustrated by being too tired	
	Feel sluggish	
	Feel a need to nap	
Cognitive Functioning	Thinking clearly	
	Difficulty concentrating	
	Difficulty forming thoughts	
	Difficulty learning	
	Difficulty remembering	
Cataplexy (NT1 only)	Avoiding triggers	• Never (0) • Rarely (1) • Sometimes (2) • Usually (3) • Always (4)
	Dropping things	
	Problems getting around	
	Cataplexy worries	
	Injury worries	
Social Activities	Activities with family and friends	
	Trouble socializing	
	Had to cancel plans	
	Limited spontaneity	
Everyday Activities	Difficulty watching TV/movie	
	Difficulty reading	
	Sleep attacks	
	Nap during day	
Everyday Responsibilities	Difficulty with work/school	
	Limited doing work	
	Difficulty with personal and household responsibilities	

Summary scores for each of the 6 domains are calculated and transformed to a standardized scale from 0 (best health) to 100 (worst health).

^*^Representative items shown; the FINI contains copyrighted items from PROMIS Health. Organization and Takeda Development Center Americas, Inc., all rights reserved.

FINI, Functional Impacts of Narcolepsy Instrument; NT1, Narcolepsy Type 1.

FINI psychometric properties and MWPC thresholds were assessed using data from 2 randomized, placebo-controlled, double-blind phase 3 clinical trials evaluating the efficacy and safety of TAK-861 (oveporexton) in people with NT1 (TAK-861-3001; NCT06470828, and TAK-861-3002; NCT06505031) (27). Patient-reported outcome populations (PRPs) were derived from the full analysis set population for each study, including all participants who gave a response to at least 1 FINI item at baseline. Except for MWPC estimation, all psychometric analyses were conducted using the pooled dataset combining PRPs from both studies. For MWPC estimation, each dataset was analyzed separately. In brief, for both studies, a screening period of up to 50 days was followed by 12 weeks of treatment in which the FINI was administered at baseline, Week 8 (Day 56), and Week 12 (Day 84). The analyses presented in this report were performed using only data from these timepoints. The TAK-861-3001 study enrolled 168 participants from 11 countries including Japan, the United States, Spain, France, Canada, Switzerland, Germany, Italy, Norway, Great Britain, and the Netherlands. The TAK-861-3002 study enrolled 105 participants from 11 countries: 3 countries (France, Italy, and Spain) were in both studies, and 8 countries (Belgium, China, Korea, Finland, Australia, Austria, Poland, and Sweden) were unique to TAK-861-3002. The English version of the FINI was translated into 30 local languages for use in the TAK-861-3001 and TAK-861-3002 studies in alignment with the ISPOR Translation and Cultural Adaptation guidelines (26), including cognitive debriefing conducted in the target language with participants with narcolepsy before finalization (8).

PROs and clinician-reported outcomes were collected in both studies. Descriptions of these COA measures, along with the psychometric analyses in which they were used, are provided in Supplementary Table 1. COAs collected at screening, baseline, Week 8, and Week 12 measured functional impacts of narcolepsy (FINI), EDS (ESS: 8-item measure of EDS, each item assesses likelihood of dozing in various situations), HRQoL (Short Form-36 Survey [SF-36]: 36 items, 8 domains), health status (EuroQol-5 Dimensions 5-Levels [EQ-5D-5L]: 5 items), severity of narcolepsy symptoms (NSS-CT: 15-item assessment of overall severity and consequences of narcolepsy symptoms), and cognitive complaints (British Columbia Cognitive Complaints Inventory – Extended [BC-CCI-E]: 9 items). Patient- and clinician-reported global severity were measured at baseline and Week 12 with the single-item Patient Global Impression of Severity (PGI-S) and Clinician Global Impression of Severity (CGI-S), and global change in patient- and clinician-reported narcolepsy symptoms were collected at Week 8 and Week 12 with the Patient Global Impression of Change (PGI-C) and Clinician Global Impression of Change (CGI-C). An e-diary for weekly cataplexy rate (WCR) was also collected daily during the week prior to baseline, Week 8, and Week 12.

### 2.2 FINI data, statistical analysis, and psychometric properties

Psychometric evaluation of the FINI v1.0 followed best practice methodologies recommended for PRO measures (28–31) to ensure that the FINI v1.0 is fit-for-purpose to assess outcome in narcolepsy clinical trials, here the efficacy of oveporexton treatment in people with NT1. Validation of the FINI focused on assessing individual item performance and domain structure, followed by evaluation of domain-level reliability, validity, and responsiveness, and derivation of MWPC thresholds for this participant population. All psychometric analyses were conducted using SAS Version 9.4 or higher, except for CFA, which was performed with MPlus (Version 8.11).

#### 2.2.1 Quality of completion

The quality of completion of the FINI items was assessed at baseline, Week 8, and Week 12. A completion rate of ≥80% for each FINI item was considered acceptable.

#### 2.2.2 Item distribution and floor/ceiling

Item distribution was assessed to determine the extent to which all available response options in the FINI were utilized. Floor and ceiling effects for each FINI item were based on the proportion of participants who responded at the highest response option (4; i.e., worst health) and the lowest response option (0; i.e., best health), respectively. Floor and ceiling effects were defined as having ≥20% of participants with item scores in the lowest or highest response categories.

#### 2.2.3 Confirmatory factor analysis

CFA was performed previously during the item development process (8) to assess the scale structure of the FINI. In this present study, a CFA model was generated using the weighted least squares mean and variance adjusted method as a nonparametric method designed specifically for ordinal measures. An acceptable CFA model fit was indicated by a comparative fit index (CFI) and non-normed fit index (NNFI) >0.90, standardized root mean residual (SRMR) and root mean square error of approximation (RMSEA) < 0.08.

#### 2.2.4 Known-groups validity

Known-groups validity was evaluated by the ability of each FINI domain to differentiate between distinct groups of participants differing by symptom severity based on the PGI-S and ESS (Normal/Mild = 0–14; Moderate = 15–17; Severe EDS = 18–24) (32, 33). The magnitude of these differences, as indicated by standardized effect sizes (SES), defined as a small difference (0.2), moderate difference (0.5), or large difference (0.8) in reference mean scores, as well as by the statistical significance of the differences, were used to assess known-groups validity.

#### 2.2.5 Convergent/divergent validity

Convergent validity was evaluated by the degree to which the FINI domain scores were associated with the domain scores of other outcome assessments measuring a similar construct, and divergent validity by the degree to which the FINI domain scores were less associated with other outcome assessments not designed to measure the same construct. The selection of the proposed anchors was based on direct feedback from the FDA Division of Clinical Outcome Assessment following a Type C meeting with the agency. Anchor measures were selected based on their conceptual relevance to the corresponding FINI domains and were subsequently evaluated using correlation analyses. Spearman correlations were calculated between the continuous FINI domain scores and continuous SF-36 domain scores, NSS-CT total score, BC-CCI-E total score, WCR, and ESS score. Polyserial correlations were calculated between the continuous FINI domain scores and categorical BC-CCI-E item and EQ-5D-5L Usual Activities domain scores. The magnitude of correlations was defined as weak (< 0.3), moderate (0.3–0.7), strong (0.7–0.9), or very strong (>0.9) (34).

#### 2.2.6 Internal consistency reliability

Internal consistency was evaluated by the extent to which individual items within each of the FINI domains were consistent with each other. Cronbach's alpha was calculated for each FINI domain to estimate internal consistency within the domain. A Cronbach's alpha value of ≥0.70 was taken to indicate acceptable internal consistency.

#### 2.2.7 Test-retest reliability

Test-retest reliability was evaluated by the degree to which domain scores were consistent among stable participants. Stable participants were defined using the PGI-S as a reference measure at screening and baseline. A two-way random-effects single-rater intraclass correlation coefficient (ICC) value ≥0.70 indicated acceptable agreement. The subpopulation included all individuals with available FINI data at both screening and baseline, with < 3 weeks between screening and baseline, and who indicated no change between these time points based on PGI-S responses.

#### 2.2.8 Responsiveness

Responsiveness was evaluated by the degree of association between change in the FINI domain scores and change in specific reference measures from baseline to both Week 8 and Week 12. The following reference measures were used: PGI-S for narcolepsy symptom severity; SF-36 Vitality Items 3 and 4 (for the Tiredness domain); BC-CCI-E Items 1, 2, 3, and 5 (Cognitive Functioning domain); NSS-CT Cataplexy Impact – Item 10 (Cataplexy domain); SF-36 Social Functioning Item 2, and NSS-CT Social/Family Life Disruption – Item 4 (Social Activities domain); SF-36 Role Physical Item 4, and EQ-5D-5L Usual Activities (Everyday Activities domain); SF-36 Role Physical Item 4, EQ-5D-5L Usual Activities, and NSS-CT Work/Activity Disruptions – Item 3 (Everyday Responsibilities domain) (17, 18, 35–38). Participants were stratified on the reference measures (e.g., markedly improved, moderately improved, minimally improved vs. no change/worsened categories).

Correlations between continuous change scores were calculated. Within-group changes were determined by the standardized response mean (SRM), calculated by dividing the mean change score between baseline and subsequent time points by the SD of change scores. Between-group SRM was calculated as the mean difference in change scores divided by the pooled SD of change. The magnitude of responsiveness, based on Cohen's recommendations, was defined as small (SRM = 0.20), moderate (0.50), and large (0.80).

### 2.3 MWPC thresholds

MWPC thresholds for the FINI were determined primarily using an anchor-based approach, supported by distribution-based methods (30, 39, 40). Two types of anchors were used: concept-specific anchors that closely matched the concept of interest of each domain, and anchors based on global impression scales (e.g., PGI-S). For each anchor, participants were initially classified according to the raw level of change on the anchor at Week 8 or Week 12, i.e., uncollapsed change. Participants were then classified into broader response groups depending on the distribution of each anchor variable, i.e., collapsed change. Anchors were deemed suitable for use in the MWPC estimation if they were at least moderately correlated with the FINI domains with a correlation coefficient of ≥|0.30| (39).

The MWPC threshold determination considered the totality of evidence (i.e., triangulation), including supportive information from meta-analytic correlation-weighted estimates of mean change by anchor category, empirical cumulative distribution function (eCDF) and probability density function (PDF) curves by anchor category, as well as distribution-based methods with the calculation of 0.5 SD and the standard error of measurement (SEM=SD^*^√[1–Cronbach's alpha]) derived from baseline data. Results from concept-specific anchor estimates were prioritized during triangulation. In addition, forest plots were used to visualize changes within each anchor category and as supportive evidence of the derived MWPC using a formal meta-analytic approach.

Both the TAK-861-3001 and TAK-861-3002 datasets were analyzed separately, with results compared to determine whether a single threshold could be derived based on results from both studies. MWPC was defined as the level of change in score that participants perceived as a meaningful improvement in their narcolepsy-specific functional impacts, as measured by the FINI.

## 3 Results

### 3.1 Study populations

A total of 167 participants with NT1 from the TAK-861-3001 study and 105 from the TAK-861-3002 study were included in this analysis. The participants had an ESS score of ≥11 at screening with 79.4% having severe EDS (ESS score ≥16). Participant demographics from the TAK-861-3001 and TAK-861-3002 studies are summarized in Table 2. Mean age was similar across both studies, and approximately half of participants were female. In these global studies, 39% of participants identified as White, 17% as Asian, with race unknown for 35%. The largest number of participants were from France (*n* = 52), Italy (*n* = 35), Spain (*n* = 33), USA (*n* = 25) and Japan (*n* = 25), with representation also from China, Belgium, Germany, Australia, Canada, Finland, Switzerland, Korea, Norway, Poland, Austria, the United Kingdom, Sweden, and the Netherlands.

**Table 2:** Participant demographics and clinical characteristics.

Characteristics		TAK-861-3001 (*N* = 167^*^)	TAK-861-3002 (*N* = 105)	Pooled (*N* = 272)
Age, years	Mean (SD)	31.4 (11.30)	30.7 (11.10)	31.1 (11.21)
Sex, *n* (%)	Female	97 (58.1)	50 (47.6)	147 (54.0)
	Male	70 (41.9)	55 (52.4)	125 (46.0)
Race, *n* (%)	White	58 (34.7)	47 (44.8)	105 (38.6)
	American Indian or Alaska Native	1 (0.6)	NA	1 (0.4)
	Asian	26 (15.6)	21 (20.0)	47 (17.3)
	Black or African American	7 (4.2)	NA	7 (2.6)
	Multiple	1 (0.6)	NA	1 (0.4)
	Not Reported	15 (9.0)	NA	15 (5.5)
	Unknown	59 (35.3)	37 (35.2)	96 (35.3)
Country, *n* (%)	**Asia Pacific**			
	Japan	25 (15.0)	NA	25 (9.2)
	China	NA	14 (13.3)	14 (5.2)
	Australia	NA	8 (7.6)	8 (2.9)
	South Korea	NA	7 (6.7)	7 (2.6)
	**Europe**			
	France	31 (18.6)	21 (20.0)	52 (19.1)
	Spain	24 (14.4)	9 (8.6)	33 (12.1)
	Italy	19 (11.4)	16 (15.2)	35 (12.9)
	Switzerland	15 (9.0)	NA	15 (5.5)
	Belgium	NA	13 (12.4)	13 (4.8)
	Germany	9 (5.4)	NA	9 (3.3)
	Norway	7 (4.2)	NA	7 (2.6)
	Finland	NA	7 (6.7)	7 (2.6)
	Poland	NA	7 (6.7)	7 (2.6)
	Netherlands	4 (2.4)	NA	4 (1.5)
	Austria	NA	2 (1.9)	2 (0.7)
	Great Britain	1 (0.6)	NA	1 (0.4)
	Sweden	NA	1 (1.0)	1 (0.4)
	**North America**			
	United States	25 (15.0)	NA	25 (9.2)
	Canada	7 (4.2)	NA	7 (2.6)
ESS (daytime sleepiness categories), *n* (%)	Lower normal (0–5)	0 (0.0)	0 (0.0)	0 (0.0)
	Higher normal (6–10)	3 (1.8)	2 (1.9)	5 (1.8)
	Mild excessive (11–12)	3 (1.8)	8 (7.6)	11 (4.0)
	Moderate excessive (13–15)	23 (13.8)	17 (16.2)	40 (14.7)
	Severe excessive (6–24)	138 (82.6)	78 (74.3)	216 (79.4)

ESS, Epworth Sleepiness Scale; FINI, Functional Impacts of Narcolepsy Instrument; NA, not available for this study; SD, standard deviation.

^*^One participant from the TAK-861-3001 study (*N* = 168) did not have FINI data at baseline.

### 3.2 FINI psychometric properties

An overview of the FINI psychometric properties supporting the FINI v1.0 as a fit-for-purpose instrument for assessing outcomes in participants with NT1 is provided in Table 3 and described in more detail below.

**Table 3:** Summary of psychometric properties of the FINI.

Psychometric Property							
Confirmatory factor analysis	Good fit statistics met thresholds, confirming the FINI 6-domain scale structure CFI: 0.96; RMSEA: 0.07; NNFI: 0.95; SRMR: 0.06						
		FINI Domain					
	Expected correlations	Tiredness	Cognitive Functioning	Cataplexy	Social Activities	Everyday Activities	Everyday Responsibilities
Known-groups validity (SES ranges)		0.24–0.94	0.09–0.84	0.13–0.67	0.095–0.90	0.39–0.77	0.14–0.89
Convergent/divergent validity (Expected vs. observed average correlation with COAs^*^)	*Small*	0.22	0.31	0.36	0.41	0.23	0.21
	*Low mod*.	0.46	0.45	0.47	0.48	0.40	0.46
	*High mod. to strong*	0.53	0.58	0.66	0.56	0.35	0.54
Internal consistency reliability (Cronbach's alpha)		0.90	0.92	0.78	0.88	0.63	0.82
Test-retest reliability (ICC)		0.82	0.79	0.85	0.81	0.80	0.85
Responsiveness to change (Correlation ranges)		−0.76 to 0.67	0.52–0.78	0.52–0.61	−0.48 to 0.60	−0.50 to 0.62	−0.54 to 0.63

CFI, comparative factor index; COA, clinical outcomes assessment; FINI, Functional Impacts of Narcolepsy Instrument; ICC, intraclass correlation coefficient; mod, moderate; NNFI, non-normed fit index; RMSEA, root mean square error of approximation; SES, standardized effect size; SRMR, standardized root mean square residual.

^*^Expected correlations: small, < 0.3; low moderate, 0.3 to < 0.5; high moderate to strong, >0.5. Averages were calculated across the domains for all observed correlations in each category of expected strength.

#### 3.2.1 Quality of completion

Excellent quality of completion was observed at all timepoints. At baseline, all the FINI items were completed by 100% of participants. A high completion rate for all items was also observed during follow-up at Week 8 (99.6%) and Week 12 (99.2%).

#### 3.2.2 Item distribution and floor/ceiling

Ceiling and floor effects for each FINI item at baseline and Week 12 are summarized in Supplementary Table 2. At baseline, floor effects were observed for 14 of 28 FINI items. The largest floor effects (>67%) were reported for the 2 nap-related items (“Feel a Need to Nap” and “Nap During Day”). At baseline, ceiling effects were observed for 4 of 28 FINI items. The largest ceiling effect reported was for the “Injury Worries” item (34.2%). At Week 12, ceiling effects were observed for almost all FINI items, indicating a high endorsement of the best response option (i.e., best health). Similar ceiling effects were observed at Week 8.

#### 3.2.3 Confirmatory factor analysis

The CFA model converged with good fit statistics (Table 3), confirming the 6-domain FINI scale structure and aligning with results from the previous version of the FINI (8), even though there were slight modifications to the items. In this analysis of phase 3 data, all metrics met or exceeded predefined CFA thresholds: CFI exceeded 0.95, the NNFI was 0.95, and the RMSEA and SRMR values were both < 0.08.

An examination of factor loadings for each FINI item within their respective domain indicated that the loadings exceeded the 0.50 threshold in all domains except the Everyday Activities domain, which contained the modified “Nap during day” (item #25) and had a factor loading of 0.47. This item was, however, retained based on the generally good global fit statistics, strong theoretical justification, and its meaningful contribution to the content validity of the FINI based on the discussions with patients and clinical experts.

#### 3.2.4 Known-groups validity

Good known-groups validity was observed for all FINI domains, especially for comparisons between severe and moderate PGI-S and ESS groups. For participants reporting severe versus moderate PGI-S scores, their FINI domain scores were significantly higher across all domains with all *P*-values < 0.0001 and large SES values ranging from 0.67–0.94 (Supplementary Figure 1A). Similarly, all the FINI domain scores were significantly higher for participants with severe versus moderate EDS, as determined by the ESS, with all *P*-values < 0.05 and moderate SES values ranging from 0.34–0.68 (Supplementary Figure 1B).

#### 3.2.5 Convergent/divergent validity

Overall, good convergent and divergent validity were demonstrated. Moderate to strong correlations were observed between the FINI domains and measures assessing similar constructs, and were generally aligned with theoretical expectations (Supplementary Table 3). The observed correlations were much larger between reference measures and the FINI domains that were conceptually aligned, supporting convergent validity (domain average of 0.54). By comparison, correlations were smaller between reference measures and the FINI domains for constructs that were not (or less) closely related, supporting divergent validity (domain average of 0.29).

The strongest correlations were between the most closely aligned constructs: FINI Tiredness and SF-36 Vitality (r = −0.68); FINI Cognitive Functioning and the BC-CCI-E total score (r = 0.81); FINI Cataplexy and NSS-CT Item 08 “Weekly Generalized Cataplexy Rate” (r = 0.50) and NSS-CT Item 10 “Work/social Affected by Cataplexy” (r = 0.66); FINI Social Activities and SF-36 Social Functioning (r = −0.56), NSS-CT Item 04 “Social Disruptions (r = 0.55), and BC-CCI-E Item 09 “Social Difficulty” (r = 0.64); and FINI Everyday Responsibilities and SF-36 Role Physical (r = −0.52), NSS-CT Item 03 “Work Disruptions” (r = 0.58), EQ-5D-5L Usual Activities (r = 0.52), and BC-CCI-E Item 07 “Job Difficulty” (r = 0.60).

#### 3.2.6 Internal consistency reliability

Good internal consistency reliability, defined as Cronbach's alpha ≥0.70, was observed for the FINI Tiredness (Cronbach's alpha = 0.90), Cognitive Functioning (0.92), Cataplexy (0.78), Social Activities (0.88), and Everyday Responsibilities (0.82) domains. The 4-item Everyday Activities domain was the only domain that did not meet the 0.70 threshold (Cronbach's alpha = 0.63).

#### 3.2.7 Test-retest reliability

Good-to-excellent test-retest reliability was observed for all FINI domains when stability was defined using patient-reported outcomes (i.e., PGI-S) (Table 4). All 6 domains met the ICC threshold (≥0.70) for test-retest reliability in the stable subpopulation with a < 3-week interval between screening and baseline.

**Table 4:** Test-retest reliability of the FINI domains.

Sample	FINI domain	Mean difference	ICC
No change in PGI-S between Screening and Baseline, subsample with < 3 weeks between Screening and Baseline **(*****N*** **=** **31)**	Tiredness	2.88	**0.82**
	Cognitive Functioning	3.55	**0.79**
	Cataplexy	−1.29	**0.85**
	Social Activities	2.21	**0.81**
	Everyday Activities	−0.81	**0.80**
	Everyday Responsibilities	1.08	**0.85**

CI, confidence interval; FINI, Functional Impacts of Narcolepsy Instrument; ICC, intraclass correlation coefficient; PGI-S, Patient Global Impression of Severity.

ICC point estimates and CI values above the 0.70 threshold are indicated in **bold**. Stability was defined using the PGI-S.

#### 3.2.8 Responsiveness

Good responsiveness of all the FINI domains was observed for PGI-S as a reference measure (Supplementary Figure 2) and all other selected reference measures (Supplementary Table 4). Correlations between change in the FINI domains and proposed reference measures all exceeded 0.4, except for the correlation at Week 8 between the FINI Everyday Responsibilities and EQ-5D-5L Usual Activities domains (r = 0.37). Generally, correlations were slightly higher at Week 12 vs. Week 8.

Greater improvement in the FINI domain scores was associated with greater improvement in the reference measures (i.e., a monotonic change). Most within-group comparisons of change from baseline were statistically significant with *P*-values < 0.05 and large effect sizes, with the within-group SRM increasing the greater the improvement in the reference measure. For the majority of comparisons in the moderately/markedly improved categories, the SRM for within-group comparison was more than twice (i.e., >1.60) the standard 0.80 for a large effect size. Most between-groups comparisons between severity categories were also statistically significant, with *P*-values < 0.05 and an average effect size of 0.69.

### 3.3 MWPC thresholds

All correlations between the FINI domain scores and prespecified anchor measures exceeded the |0.30| threshold for both the TAK-861-3001 and TAK-861-3002 studies (Supplementary Table 5). Some correlations far exceeded the minimum threshold, for example, between the Tiredness domain and PGI-C and SF-36 Vitality Item 04 “Tired” (both studies r > |0.70|); the Cognitive Functioning domain and BC-CCI-E Items 02 “Poor Concentration” and 05 “Slow Thinking” (both studies r > |0.70|); and the Everyday Responsibilities domain and NSS-CT Item 03 “Work Disruptions” (both studies r > |0.55|). As a result, all potential anchor measures were taken forward for estimating MWPC thresholds. Subsequent analyses are discussed for collapsed anchor categories.

Anchor-based examination of change in the FINI domain scores across anchor categories showed a general trend of monotonic improvement in the FINI domain scores as anchor scores indicated improvement. As illustrated by the data for the PGI-S anchor for each domain, which generally had a high moderate correlation with the FINI domains (see Supplementary Table 6), the mean and median values typically demonstrated a stepwise progression through the categories of anchor improvement. Some overlap was observed between the 95% CI values of mean change scores between the “Much Improved” and “Improved” categories, reflecting the relatively small sample size of the “Much Improved” category. However, importantly, there were very few overlaps between the “Minimally Improved” and the “Stable/Worsened” categories (bold values in Supplementary Table 6). Specifically, the FINI domain scores in the “Minimally Improved” anchor categories were distinctly improved relative to those for the “Stable/Worsened” categories, as demonstrated by non- or minimally overlapping 95% CIs coupled with significant differences across categories. Similar findings were also observed across other predefined anchors within each FINI domain and were consistent for both of the phase 3 studies. Consequently, the “Minimally Improved” group was used to estimate the MWPC thresholds.

Data from the Minimally Improved/1-point change category for each anchor at Week 12 by FINI domain are summarized in Supplementary Table 7, including the simple means of the extracted data with simple correlation-weighted mean estimates and correlation-weighted 95% CIs. Averages are shown for the global impression and concept-specific anchors separately, as well as for all anchors combined. Although there was a slight CI difference between studies for some domains, the mean and median values for the “Minimal Improvement” groups were generally consistent between the TAK-861-3001 and TAK-861-3002 studies for each of the domains, and between the simple mean and weighted mean estimates. On triangulation, the concept-specific anchor correlation-weighted estimates were prioritized, given their conceptual alignment with the FINI domains.

The eCDF curves allowed assessment of the degree to which the anchor categories were separated by the FINI domain scores. Graphical eCDF/PDF curves provided supportive evidence for estimating MWPC thresholds using the “Minimally Improved” category for both the TAK-861-3001 and TAK-861-3002 studies. An illustrative example for the Tiredness domain at Week 12 is shown in Figure 1. There was clear separation among all categories: “Stable/Worsened” vs. “Minimally Improved” vs. “Improved” vs. “Much Improved.” The PDF curves allowed a graphical representation of the central tendency and variability of the FINI domain score within each anchor category. The PDF curves indicate some overlap between the “Improved” and “Much Improved” categories, with the best differentiation observed between the “Stable/Worsened” and “Minimally Improved” categories.

**Figure 1:**
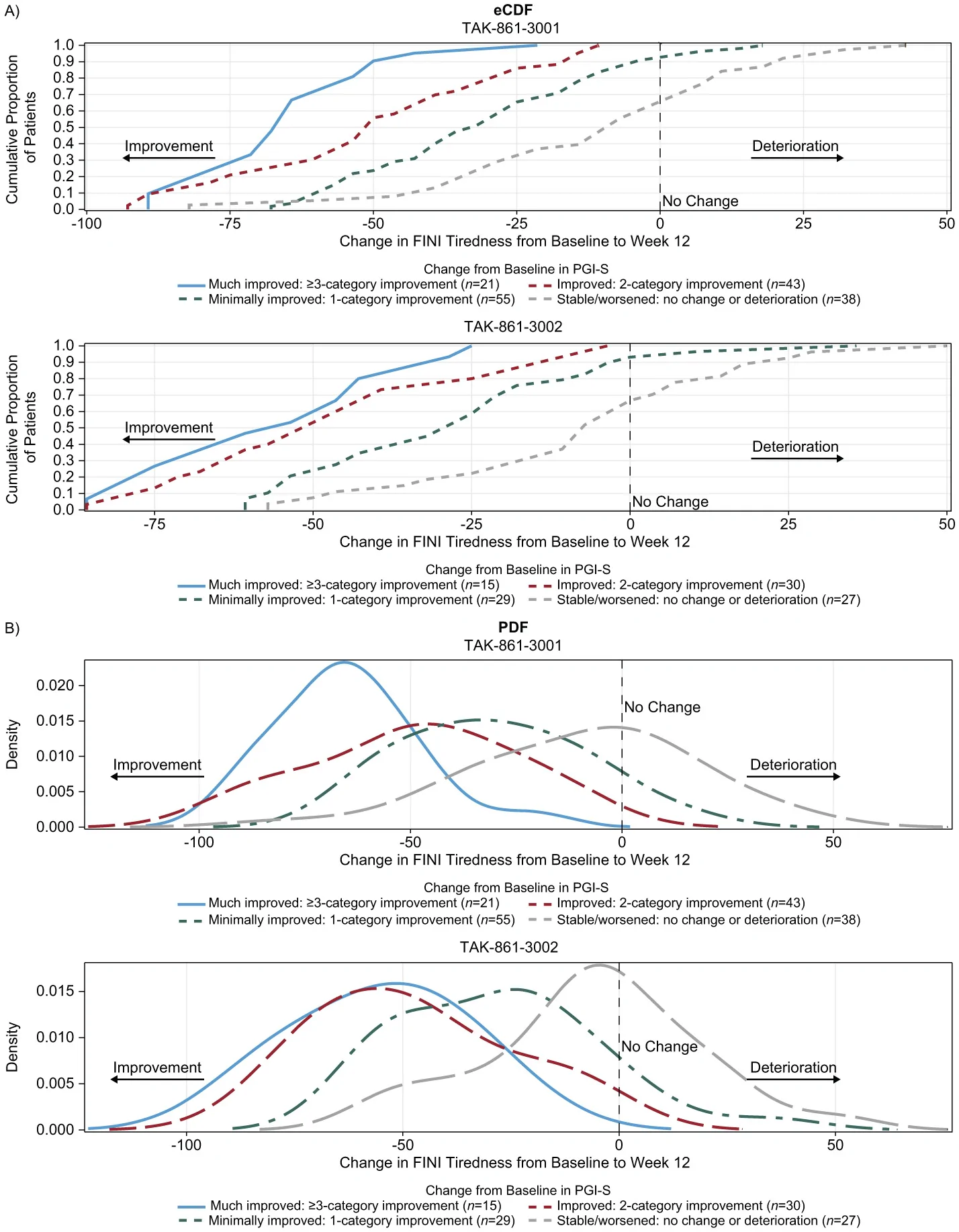
Illustrative **(A)** empirical cumulative distribution function (eCDF) and **(B)** probability density function (PDF) curves from the TAK-861-3001 and TAK-861-3002 studies. Results for change from baseline in the FINI Tiredness domain at Week 12 are shown. FINI, Functional Impacts of Narcolepsy Instrument; PGI-S, Patient Global Impression of Severity.

For the TAK-861-3001 study, forest plots are summarized for all prespecified anchors, for each domain in Supplementary Figure 3. Similar to the pattern observed with the PDF curves, the forest plots demonstrated overlap between the “Improved” and “Much Improved” categories, but strong differentiation between the “Stable/Worsened” and “Minimally Improved” categories, especially for the overall estimate for each anchor category, weighted by the correlation with the collapsed anchor. Similar forest plots were observed for the TAK-861-3002 study.

The anchor-based analyses were also supported by distribution-based analyses. SEM ranged from ≥6–12, and 0.5 SD ranged from ≥8–14 (Table 5). As such, a change of 14 across each domain was considered to be above measurement error (i.e., derived MWPC thresholds should meet or exceed this value).

**Table 5:** Distribution-based method to evaluate FINI measurement variability.

FINI domain	*n*	SD	0.5 SD	SEM^*^
TAK-861-3001 study				
Tiredness	167	22.23	11.11	6.82
Cognitive Functioning	167	26.23	13.12	7.72
Cataplexy	167	22.15	11.08	10.75
Social Activities	167	26.82	13.41	9.31
Everyday Activities	167	18.89	9.45	11.25
Everyday Responsibilities	167	25.77	12.89	10.31
TAK-861-3002 study				
Tiredness	105	20.22	10.11	6.55
Cognitive Functioning	105	25.51	12.76	7.31
Cataplexy	105	24.26	12.13	10.64
Social Activities	105	26.32	13.16	9.30
Everyday Activities	105	17.51	8.75	10.89
Everyday Responsibilities	105	23.74	11.87	11.03

^*^Derived as SEM = SD √ (1-Cronbach's alpha).

FINI, Functional Impacts of Narcolepsy Instrument; MWPC, meaningful within-patient change; SD, standard deviation; SEM, standard error of measurement.

MWPC was estimated as a single, rounded point estimate to reflect the actual FINI domain scores, as well as a range of estimates (Supplementary Table 7). Threshold values were primarily estimated from the anchor-based analyses and supported by the distribution-based analysis and examination of eCDF/PDF curves and forest plots. Given the similarity of results across the totality of evidence, a single set of thresholds across both studies was derived. The point estimate was taken as the mid-point of the range and the final MWPC thresholds were: −35 (−30 to −40) for both Tiredness and Everyday Activities, −30 (−25 to −35) for both Cognitive Functioning and Everyday Responsibilities, −25 (−20 to −30) for Social Activities, and −20 (−15 to −25) for Cataplexy.

## 4 Discussion

The FINI is a 28-item, 6-domain narcolepsy-specific measure developed with feedback from people with narcolepsy, clinical outcome scientists, and clinical experts treating people with narcolepsy (8). The FINI captures the symptoms and functional impairments experienced by people with NT1 and NT2. This study provides evidence for strong measurement properties of the FINI in NT1, with results generally meeting or exceeding required standards for psychometric properties for a PRO. The 6-domain structure of the FINI was confirmed on CFA, using a different dataset from the initial CFA supporting 6 separate domains. Each of the 6 domains generally demonstrated good validity (known groups and convergent/divergent), reliability (internal consistency, test-retest), and responsiveness.

The FINI fills an important gap in the assessment of narcolepsy by providing a responsive outcome measure for evaluating the effect of new treatments on day-to-day functioning. It complements established and new sleep-related instruments in narcolepsy trials, including the ESS (9), and other measures of broader functioning, quality of life, and disease burden, such as PROM-CDH (19), and NSS-CT (17, 18). Furthermore, these tools together offer an opportunity to continue to expand understanding of the patient experience by going beyond the pentad symptoms, thus enabling further research on the broader impact of narcolepsy on daily functioning and overall HRQoL (41).

Data completeness of the FINI at baseline, Week 8, and Week 12 was >99%, indicating a very high level of acceptability of the FINI questionnaire in a global population of participants with NT1. Research indicates that high PRO completion rates are achieved when PROs are designed to minimize patient burden, which includes items that are relevant to the patient experience (42). The high completion rate of the FINI underscores the ease of use of the FINI, and supports the reliability of psychometric results.

Several features of the FINI highlight its utility as an assessment that is well suited for determining treatment effects in a clinical trial setting. At baseline, floor effects were observed for half of the FINI items, indicating that the FINI is representative of disease-specific impacts. More floor than ceiling effects for the FINI items were observed, suggesting the FINI overall measures disease-specific impacts at baseline when participant conditions are expected to be poor. Given that participants had an ESS score of ≥11 at screening and 79.4% had severe EDS (ESS score ≥16), these floor effects may be at least partly artifactual, reflecting the severity of the enrolled population; a sample with a broader range of symptom severity would likely have produced a more balanced distribution across FINI item response categories. During follow-up visits at Week 8 and Week 12, ceiling effects were observed for almost all FINI items, consistent with an investigational drug effect. Responsiveness of FINI scores was exceptional, indicating that the FINI is highly sensitive to detecting change in response to treatment. The correlations between the FINI domains and all reference measures exceeded 0.35. Notably, monotonic improvement in all FINI domain scores across all reference measures was observed, with the effect sizes detecting change from baseline (within-group SRM) being large for most analyses. Furthermore, within the Moderately/Markedly Improved categories, the effect size was frequently more than double the standard for large effect size. These results highlight the ability of the FINI to detect a treatment effect and the utility of the FINI for detecting changes in functional impacts over time.

The FINI represents a PRO that aligns with the Food and Drug Administration Patient-focused Drug Development (FDA-PFDD) guidance for developing fit-for-purpose clinical outcome assessments (43). The methodology outlined in the PFDD guidance provides more assurance that, if a treatment effect is identified by the FINI, the results are due to direct benefits of treatment, rather than spurious results associated with poor psychometric properties. In particular, validity and reliability of the FINI were very strong. Good known-groups validity supports the use of the FINI as a sensitive measure differentiating individuals with varying levels of symptom severity. Both convergent/divergent validity of the FINI were also supported. Reliability of the FINI was excellent, as demonstrated by strong internal consistency and test-retest reliability results. Good-to-excellent test-retest reliability demonstrates that the FINI performs well and produces stable results when administered repeatedly over time in stable conditions.

Good internal consistency indicates that the patient-centered approach for deriving FINI domain scores resulted in items that accurately reflect the concept measured by each domain. While the internal consistency of 5 domains met pre-specified thresholds, the Everyday Activities domain demonstrated lower internal consistency (Cronbach's α = 0.63) than the pre-specified threshold of 0.70. These findings suggest greater heterogeneity among the concepts captured within the domain. However, the domain was intentionally designed to assess a broad range of everyday functional impacts reported by patients, and all items were retained to preserve content validity established through qualitative patient research and expert review (8). Therefore, the Everyday Activities domain may be associated with greater measurement error, which should be taken into account when powering future studies.

Interpretation of PRO data is greatly enhanced when there is an estimate of clinically meaningful change (30, 40, 44). MWPC estimates, defined as the threshold for change in domain scores that patients perceive as beneficial, facilitate interpretation of the magnitude of effect and are the preferred method to be utilized to identify the proportion of responders in each treatment group. However, methods for MWPC have been evolving over the past decade and few studies have operationalized the recent methods proposed by the FDA (30, 45). MWPC thresholds were derived for all 6 FINI domains to aid in interpretation of FINI scores with support from eCDF and PDF curves, which showed clear separation in the expected direction between anchor groups across all FINI domains. The current analysis also demonstrates a novel empirical method to aid in derivation of MWPC values by leveraging forest plots to derive overall meta-analytic estimates (46). The forest plots illustrate consistent performance across anchors in differentiating FINI change scores between people with stable and minimally improved functional impacts. Estimates using both global and concept-specific anchors were comparable, and these were supported by the meta-analytic overall estimates. In addition, the final MWPC thresholds derived from the phase 3 study data were consistent with those reported previously for an earlier draft of the FINI utilizing phase 2 trial data (24). The novel meta-analytic method illustrated here can be used in future research to analyze multiple anchors with distinct relationships to the endpoint (i.e., correlation strength) (46). These MWPC thresholds for improvement will aid in the interpretation of FINI results. For example, the MWPC threshold values can be used in a responder analysis to determine the percentage of participants in each treatment group who had a meaningful change (i.e., having a change that met or exceeded the MWPC threshold) for each FINI domain.

The FINI offers advantages for clinical settings beyond its utility in a clinical trial setting, with potential to support symptom recognition and communication between health care providers (HCPs) and people with narcolepsy type 1. As a simple symptom-tracking tool and resource, it can help people with narcolepsy distinguish functional impacts that are most relevant to their daily lives. The modular structure (i.e., 6 separate domains) can facilitate shared treatment decisions between HCPs and patients, for example, to track progress in specific domain(s) of relevance to people with NT1.

A limitation of this study is the inclusion of data from clinical trial participants only, which limits the generalizability of the results to populations outside the trial, including people with NT1 with less severe EDS. However, early development of the FINI (including content validity and item selection) leveraged data from a broader narcolepsy population that included seven observational studies and a Phase 2 clinical trial (8). In addition, good psychometric properties were previously demonstrated for an earlier version of the FINI in both an observational study and a Phase 2 clinical trial (24). Because the clinical trials required an ESS score ≥11 at study entry, people with well-controlled EDS were underrepresented in the psychometric evaluation sample. Consequently, the observed floor effects may be artificial and may not be fully generalizable to patients with lower levels of daytime sleepiness who continue to experience other functional impacts of narcolepsy. Additional evaluation in broader real-world populations is warranted, including pediatric populations, and individuals with systemic, neurologic, or psychiatric comorbidities. Furthermore, dedicated measures of depression or anxiety were not included in the psychometric evaluation, reflecting decisions made during clinical development, as people with severe depression were excluded from the clinical trials. Consequently, the ability to evaluate relationships between FINI scores and psychiatric comorbidity was limited. Future studies should further evaluate the association between FINI and mood-related outcomes in broader narcolepsy populations. Questionnaire completion time, time-of-day effects, and potential item-order effects were not evaluated in the current study. While the FINI uses a 7-day recall period and is therefore less dependent on momentary cognitive state than performance-based measures, the potential influence of transient sleepiness, cognitive impairment, or survey-order effects on responses cannot be ruled out. Additionally, while the FINI for NT2 populations intends to include the 5 domains relevant to NT2 (i.e., all FINI domains except for the Cataplexy domain), full psychometric results are not available. Psychometric evaluation of the FINI for NT2 (without the Cataplexy domain) and IH populations will be needed to determine suitability for these populations. This expanded use for all CDH populations can facilitate evaluation of treatment effects in clinical trials, as well as other clinical settings. Finally, as a recently developed instrument, psychometric validation data are currently limited for the FINI; further external validation studies would be beneficial.

These findings demonstrate that the FINI is a robust measure, with good reliability and validity, for assessing the functional impacts experienced by people with narcolepsy on outcomes that are meaningful and important to them. The strength of these findings suggest that the FINI is not only a sensitive PRO measure for the clinical trial setting, but could also be utilized in clinical practice to support shared decision-making, alongside current conventional measures, to ensure the full scope of narcolepsy's disease-specific impact is captured.

## Data Availability

The datasets presented in this article are not readily available because they will be made available on suitable application only. The datasets, including the redacted study protocol, redacted statistical analysis plan, and individual participant data supporting the results of the study, will be made available after the publication of study results within three months from initial request to researchers who provide a methodologically sound proposal. The data will be provided after de-identification, in compliance with applicable privacy laws, data protection and requirements for consent and anonymization. Requests to access the datasets should be directed to the corresponding author.
