# Supplementary material for: Psychometric properties of the novel patient-reported Functional Impacts of Narcolepsy Instrument (FINI) using data from phase 3 clinical trials in narcolepsy type 1
Source: Front Neurol. 2026 Oct 15;17:1884901. doi: 10.3389/fneur.2026.1884901 (PMC13717241; doi:10.3389/fneur.2026.1884901)
Supplement: Supplementary file 1 [file Data_Sheet_1.docx]

Supplementary Material

# Supplementary Figures and Tables

**Supplementary Figure 1**. Known-groups validity of FINI domains (LS means with SE bars) at baseline based on (A) PGI-S and (B) ESS categories of severity.

ANOVA, analysis of variance; ESS, Epworth Sleepiness Scale; FINI, Functional Impacts of Narcolepsy Instrument; LS, least squares; PGI-S, Patient Global Impression of Severity; SE, standard error; SES, standardized effect size.

LS mean and SE are derived from an ANOVA. The 2-sided *P*-value shows the difference in LS mean scores between adjacent groups (i.e., in comparison to preceding category). The SES is calculated by dividing the difference in scores between consecutive groups by the pooled group SD. *For PGI-S: *P*-value not reported for “None” due to *n* = 1, *P*-value for “Severe” reflects differences versus “Moderate”, and *P*-value for “Moderate” reflects differences versus “Mild”. For ESS: *P-*value for “Severe” reflects differences versus “Moderate”, and *P*-value for “Moderate” reflects differences versus “Normal to Mild”.

**Supplementary Figure 2**. Responsiveness of the FINI domains (mean change with SD bars) relative to change in PGI-S from baseline to (A) Week 8 and (B) Week 12.

ANOVA, analysis of variance; FINI, Functional Impacts of Narcolepsy Instrument; LS, least squares; PGI-S, Patient Global Impression of Severity; SD, standard deviation; SRM, standardized response mean.

Correlations (r) provided are between change in FINI domain scores and change in reference measures from Baseline to Week 8 and Week 12. For within groups, SRM is calculated by dividing the mean change score between Baseline and the subsequent time point by the SD of the change score. For between groups, the SRM is calculated by dividing the difference in change scores between consecutive groups by the pooled change SD. SRM values: small change = 0.2, moderate change = 0.5, large change = 0.8. The *P*-value for each individual change group is derived from a paired *t*-test assessing the difference in the same group over time (for “within-group” comparisons). The *P*-value for the comparisons to the prior (less improved) adjacent severity group is derived from select pairwise comparisons (independent samples *t*-test) under ANOVA to assess difference in score change between groups (for “between-group” comparisons). *P*-values: **P*<0.05, ***P*<0.01, ****P*<0.001.

**Supplementary Figure 3.** Fixed-effects summary of anchor-based estimates of meaningful change for the FINI domain scores (mean with 95% CI bars) at Week 12 using all anchors (TAK-861-3001, *N* = 167).

BC-CCI-E, British Columbia Cognitive Complaints Inventory – Extended; CGI-C, Clinician Global Impression of Change; CGI-S, Clinician Global Impression of Severity; CI, confidence interval; EQ-5D-5L, EuroQol 5-Dimension 5-Level; FINI, Functional Impacts of Narcolepsy Instrument; MWPC, meaningful within-patient change; NSS-CT, Narcolepsy Severity Scale for Clinical Trials; PGI-C, Patient Global Impression of Change; PGI-S, Patient Global Impression of Severity; SF-36, Short Form-36 Health Survey.

**Supplementary Table 1.** Descriptions of the clinical outcomes assessment measures used in this analysis

| Measure | Description | Scoring | Psychometric property assessed |
| --- | --- | --- | --- |
| Functional Impacts of Narcolepsy Instrument (FINI) (1) | - A narcolepsy-specific, 28-item PRO assessing the functional impacts of narcolepsy with a 7-day recall period | - All items use a 5-point response scale (scored 0 to 4) | - Target PRO measure for which psychometric properties were generated |
| Epworth Sleepiness Scale (ESS) (2) | - A subjective, self-administered scale measuring excessive daytime sleepiness | - 8 item scores are summed for an overall score of 0 to 24 (scores below 11 are within the normal range) - Response options: 0 = “would never doze”, 1 = “slight chance of dozing”, 2 = “moderate chance of dozing”, and 3 = “high chance of dozing” | - Known-groups validity and convergent/divergent validity of FINI domains |
| Patient Global Impression Scales (PGI-S and PGI-C) (3) | - A PGI rating scale that captures participant assessments of global treatment effects on overall narcolepsy symptoms | - PGI-S: Symptom severity at time of assessment rated on 5-point scale ranging from “None” to “Very severe” - PGI-C: Participant perception of improvement in overall symptoms relative to baseline rated on a 7-point scale ranging from “Very much improved” to “Very much worse” | - PGI-S: used to define stable participants when assessing test-retest reliability, known-groups validity, and responsiveness of FINI domains - PGI-S and PGI-C: Used as prespecified anchors to estimate MWPC thresholds for FINI domains |
| Clinician Global Impression Scales (CGI-S and CGI-C) (4) | - A CGI rating scale that captures clinician assessments of global treatment effects on overall narcolepsy symptoms | - CGI-S: Symptom severity at time of assessment rated on a 7-point scale ranging from “Normal, not at all ill” to “Among the most extremely ill patients” - CGI-C: Clinician perception of improvement in overall symptoms relative to baseline rated on a 7-point scale ranging from “Very much improved” to “Very much worse” | - Used as prespecified anchors to estimate MWPC for FINI domains |
| Short Form-36 (SF-36) v2, acute (5) | - A 36-item, self-reported measure of health-related quality-of-life in 8 domains with a 1-week recall period | - Each domain is scored from 0–100 with higher scores indicating less disability | - Role Physical, Social Functioning, and Vitality domains were used to assess convergent/divergent validity - Specific items from these domains were used to assess responsiveness of the FINI and as prespecified anchors to estimate MWPC for specific FINI domains |
| Narcolepsy Severity Scale for Clinical Trials (NSS-CT) (6) | - A 15-item, self-reported scale evaluating the severity and consequences of narcolepsy symptoms (sleepiness, cataplexy, sleep paralysis, hallucinations, disrupted nocturnal sleep) with a 1-week recall period | - The total score ranges from 0 to 57 with higher scores indicating more severe disease | - NSS-CT total scores and specific items were used as reference measures for assessing convergent/divergent validity - Specific items were also selected as reference measures for assessing responsiveness or for estimating MWPC for specific FINI domains |
| EuroQol-5 Dimension 5-Level (EQ-5D-5L) (7) | - A self-reported, generic measure of health status in 5 dimensions (mobility, self-care, usual activities, pain/discomfort, and anxiety/depression) | - Each item has 5 levels: 1 = “no problems”, 2 = “slight problems”, 3 = “moderate problems”, 4 = “severe problems”, and 5 = “extreme problems” | - Usual Activities item was used to assess convergent/divergent validity and responsiveness of FINI domains, and as a prespecified anchor for estimating MWPC for specific FINI domains |
| British Columbia Cognitive Complaints Inventory – Extended (BC-CCI-E) (8) | - A 6-item measure assessing problems with concentration, memory, expressing thoughts, word finding, slow thinking, and difficulty solving problems over the previous 7 days - The expanded 9-item version includes items related to how symptoms affect difficulties doing job functions, relationships with family and friends, and enjoying social or recreational activities or hobbies | - Each item is evaluated on a 4-point scale with the following response options: 0 = “not at all”, 1 = “some”, 2= “quite a bit”, and 3= “very much” - For the 3 extended questions, participants circle responses ranging from “false, not at all true”, “slightly true”, “mainly true”, to “very true” | - BC-CCI-E total score and specific items were used to assess convergent/divergent validity of FINI domains - Specific items were also used to assess responsiveness of FINI domains and as prespecified anchors to estimate MWPC for select FINI domains |
| Weekly Cataplexy Rate (WCR) | - Daily e-diary recording partial or complete episodes of cataplexy | - WCR is calculated every 2 weeks as the total number of events per week, with adjustment for missing diary days | - 50% decrease in WCR was selected as a minimal meaningful change for the WCR anchor when estimating MWPC for the FINI Cataplexy domain |

BC-CCI-E, British Columbia Cognitive Complaints Inventory – Extended; EQ-5D-5L, EuroQol-5 Dimension 5-Level; ESS, Epworth Sleepiness Scale; FINI, Functional Impacts of Narcolepsy Instrument; MWPC, meaningful within-patient change; NSS-CT, Narcolepsy Sleepiness Scale for Clinical Trials; PRO, patient-reported outcome; SF-36, Short Form-36 Health Survey; WCR, weekly cataplexy rate.

**References**

1. Savva Y, Romero H, Crawford S, Howerter A, McPherson P, Doll H, et al. Development and content validity of the Functional Impacts of Narcolepsy Instrument: a novel patient-reported outcome measure for narcolepsy type 1 and type 2. *Sleep.* (2026) 49:zsag127. doi: 10.1093/sleep/zsag127

2. Johns MW. A new method for measuring daytime sleepiness: The Epworth Sleepiness Scale. *Sleep.* (1991) 14:540–45. doi: 10.1093/sleep/14.6.540

3. MAPI Research Trust. *Patient Global Impressions scale - Change, Improvement, Severity (PGI-C, PGI-I, PGI-S)* [Online]. Available: <https://eprovide.mapi-trust.org/instruments/patient-global-impressions-scale-change-improvement-severity> (Accessed December 12 2025).

4. MAPI Research Trust. *Clinical Global Impressions scale - Improvement, Severity, Change and Efficacy (CGI-I, CGI-S, CGI-C and CGI-E).* [Online]. Available: <https://eprovide.mapi-trust.org/instruments/clinical-global-impressions-scale-improvement-severity-change-and-efficacy> (Accessed December 12 2025).

5. Ware JE, Kosinski M, Bjorner J, Turner-Bowker D, Gandek B, Maruish M (2007). *User’s Manual for the SF-36v2 Health Survey.* Lincoln, RI: QualityMetric Incorporated.

6. Dauvilliers Y, Beziat S, Pesenti C, Lopez R, Barateau L, Carlander B, et al. Measurement of narcolepsy symptoms: The Narcolepsy Severity Scale. *Neurology.* (2017) 88:1358–65. doi: 10.1212/WNL.0000000000003787

7. EuroQol Research Foundation. *EQ-5D-5L* [Online]. Available: <https://euroqol.org/information-and-support/euroqol-instruments/eq-5d-5l/> (Accessed December 12 2025).

8. Iverson GL, Lam RW. Rapid screening for perceived cognitive impairment in major depressive disorder. *Ann Clin Psychiatry.* (2013) 25:135–40. doi: 10.1177/104012371302500208

**Supplementary Table 2.** Item distribution and ceiling and floor effects for the FINI at baseline and Week 12

|  | Baseline (*N*=272) | | Week 12 (*N*=260) | | |
| --- | --- | --- | --- | --- | --- |
| **FINI Item** | **Ceiling** | **Floor** | **Ceiling** | **Floor** |  |
| 1. Feel fatigued | 9 (3.3%) | **67 (24.6%)** | **71 (27.5%)** | 18 (7.0%) |  |
| 2. Tired | 2 (0.7%) | **96 (35.3%)** | 44 (17.1%) | 22 (8.5%) |  |
| 3. Trouble starting things | 15 (5.5%) | **59 (21.7%)** | **95 (36.8%)** | 16 (6.2%) |  |
| 4. Trouble finishing things | 10 (3.7%) | **66 (24.3%)** | **95 (36.8%)** | 14 (5.4%) |  |
| 5. Frustrated by being too tired | 16 (5.9%) | **95 (34.9%)** | **103 (39.9%)** | 29 (11.2%) |  |
| 6. Feel sluggish | 14 (5.1%) | **65 (23.9%)** | **87 (33.7%)** | 17 (6.6%) |  |
| 7. Feel a need to nap | 0 (0.0%) | **187 (68.8%)** | 45 (17.4%) | 50 (19.4%) |  |
| 8. Thinking clearly | 29 (10.7%) | 34 (12.5%) | **120 (46.5%)** | 9 (3.5%) |  |
| 9. Difficulty concentrating | 12 (4.4%) | 52 (19.1%) | **101 (39.1%)** | 19 (7.4%) |  |
| 10. Difficulty forming thoughts | 42 (15.4%) | 23 (8.5%) | **127 (49.2%)** | 10 (3.9%) |  |
| 11. Difficulty learning | 38 (14.0%) | 34 (12.5%) | **131 (50.8%)** | 13 (5.0%) |  |
| 12. Difficulty remembering | 47 (17.3%) | 35 (12.9%) | **97 (37.6%)** | 15 (5.8%) |  |
| 13. Avoiding triggers | 30 (11.0%) | 38 (14.0%) | **87 (33.7%)** | 16 (6.2%) |  |
| 14. Dropping things | **65 (23.9%)** | 17 (6.3%) | **144 (55.8%)** | 1 (0.4%) |  |
| 15. Problems getting around | **82 (30.1%)** | 16 (5.9%) | **162 (62.8%)** | 4 (1.6%) |  |
| 16. Cataplexy worries | 38 (14.0%) | **61 (22.4%)** | **102 (39.5%)** | 18 (7.0%) |  |
| 17. Injury worries | **93 (34.2%)** | 27 (9.9%) | **160 (62.0%)** | 8 (3.1%) |  |
| 18. Activities with friends and family | 45 (16.5%) | 33 (12.1%) | **127 (49.2%)** | 8 (3.1%) |  |
| 19. Trouble socializing | 33 (12.1%) | 38 (14.0%) | **128 (49.6%)** | 12 (4.7%) |  |
| 20. Had to cancel plans | **61 (22.4%)** | 24 (8.8%) | **147 (57.0%)** | 2 (0.8%) |  |
| 21. Limited spontaneity | 31 (11.4%) | 48 (17.6%) | **110 (42.6%)** | 11 (4.3%) |  |
| 22. Difficulty watching TV/movie | 13 (4.8%) | **62 (22.8%)** | **105 (40.7%)** | 17 (6.6%) |  |
| 23. Difficulty reading | 21 (7.7%) | **81 (29.8%)** | **99 (38.4%)** | 21 (8.1%) |  |
| 24. Sleep attacks | 21 (7.7%) | **60 (22.1%)** | **109 (42.2%)** | 14 (5.4%) |  |
| 25. Nap during day | 0 (0.0%) | **184 (67.6%)** | 40 (15.5%) | **57 (22.1%)** |  |
| 26. Difficulty with work/school | 21 (7.7%) | **59 (21.7%)** | **109 (42.2%)** | 20 (7.8%) |  |
| 27. Limited doing work | 20 (7.4%) | **78 (28.7%)** | **112 (43.4%)** | 25 (9.7%) |  |
| 28. Difficulty with personal and household responsibilities | 21 (7.7%) | 35 (12.9%) | **101 (39.1%)** | 11 (4.3%) |  |

|  | <15%. |
| --- | --- |
|  | 15–30%. |
|  | 30–45%. |
|  | 45–60%. |
|  | >60%. |

FINI, Functional Impacts of Narcolepsy Instrument.

A floor effect is evaluated at the point on the response scale wherein the subject had the worst outcome (i.e., greatest disease severity) measured by the scale (4 = Very much/Always), whereas a ceiling effect is evaluated at the point on the response scale wherein the subject has the best outcome (i.e., least disease severity) measured by the scale (0 = Not at all/Never). **Bolded** **values** indicate those greater than or equal to the benchmark for identifying floor and ceiling effects (i.e., ≥20%).

**Supplementary Table 3**. Expected and observed relationships between the FINI domains and reference measures at baseline

|  |  | Strength of Correlation | | |  |
| --- | --- | --- | --- | --- | --- |
| **FINI Domain** | **Reference Measure** | **Expected** | **Observed** | **Observed vs. Expected** | |
| **Tiredness** | SF-36 Role Physical | ++ | -0.429 | As expected |  |
|  | SF-36 Social Functioning | ++ | -0.371 | As expected |  |
|  | SF-36 Vitality | **+++** | **-0.679** | As expected |  |
|  | NSS-CT Total Score | ++ | 0.472 | As expected |  |
|  | NSS-CT Item 03: Work Disruptions | ++ | **0.524** | Larger |  |
|  | NSS-CT Item 04: Social Disruptions | ++ | **0.534** | Larger |  |
|  | NSS-CT Item 08: Weekly Generalized Cataplexy Rate | + | 0.218 | As expected |  |
|  | NSS-CT Item 10: Work/Social Affected by Cataplexy | ++ | 0.337 | As expected |  |
|  | EQ-5D-5L Usual Activities | ++ | 0.472 | As expected |  |
|  | BC-CCI-E Total Score | ++ | **0.517** | Larger |  |
|  | BC-CCI-E Item 07: Job Difficulty | ++ | 0.454 | As expected |  |
|  | BC-CCI-E Item 08: Relationship Difficulty | ++ | 0.462 | As expected |  |
|  | BC-CCI-E Item 09: Social Difficulty | ++ | **0.501** | Larger |  |
|  | Weekly Cataplexy Rate | + | 0.213 | As expected |  |
|  | ESS Total Score | **+++** | 0.371 | Smaller |  |
| **Cognitive Functioning** | SF-36 Role Physical | **+** | -0.418 | Larger |  |
|  | SF-36 Social Functioning | ++ | -0.401 | As expected |  |
|  | SF-36 Vitality | ++ | **-0.539** | Larger |  |
|  | NSS-CT Total Score | ++ | 0.476 | As expected |  |
|  | NSS-CT Item 03: Work Disruptions | ++ | 0.482 | As expected |  |
|  | NSS-CT Item 04: Social Disruptions | ++ | **0.548** | Larger |  |
|  | NSS-CT Item 08: Weekly Generalized Cataplexy Rate | + | 0.280 | As expected |  |
|  | NSS-CT Item 10: Work/Social Affected by Cataplexy | ++ | 0.392 | As expected |  |
|  | EQ-5D-5L Usual Activities | ++ | 0.436 | As expected |  |
|  | BC-CCI-E Total Score | **+++** | **0.812** | As expected |  |
|  | BC-CCI-E Item 07: Job Difficulty | **+++** | **0.543** | As expected |  |
|  | BC-CCI-E Item 08: Relationship Difficulty | **+++** | 0.479 | Smaller |  |
|  | BC-CCI-E Item 09: Social Difficulty | **+++** | 0.499 | Smaller |  |
|  | Weekly Cataplexy Rate | + | 0.220 | As expected |  |
|  | ESS Total Score | ++ | 0.327 | As expected |  |
| **Cataplexy** | SF-36 Role Physical | + | -0.398 | Larger |  |
|  | SF-36 Social Functioning | + | -0.439 | Larger |  |
|  | SF-36 Vitality | + | -0.279 | As expected |  |
|  | NSS-CT Total Score | ++ | **0.620** | Larger |  |
|  | NSS-CT Item 03: Work Disruptions | + | 0.342 | Larger |  |
|  | NSS-CT Item 04: Social Disruptions | + | 0.404 | Larger |  |
|  | NSS-CT Item 08: Weekly Generalized Cataplexy Rate | ++ | **0.504** | Larger |  |
|  | NSS-CT Item 10: Work/Social Affected by Cataplexy | **+++** | **0.658** | As expected |  |
|  | EQ-5D-5L Usual Activities | ++ | 0.291 | Smaller |  |
|  | BC-CCI-E Total Score | + | 0.359 | Larger |  |
|  | BC-CCI-E Item 07: Job Difficulty | + | 0.303 | Larger |  |
|  | BC-CCI-E Item 08: Relationship Difficulty | + | 0.306 | Larger |  |
|  | BC-CCI-E Item 09: Social Difficulty | + | 0.383 | Larger |  |
|  | Weekly Cataplexy Rate | + | 0.449 | Larger |  |
|  | ESS Total Score | + | 0.276 | As expected |  |
| **Social Activities** | SF-36 Role Physical | + | -0.477 | Larger |  |
|  | SF-36 Social Functioning | **+++** | **-0.563** | As expected |  |
|  | SF-36 Vitality | ++ | -0.445 | As expected |  |
|  | NSS-CT Total Score | ++ | **0.514** | Larger |  |
|  | NSS-CT Item 03: Work Disruptions | ++ | 0.436 | As expected |  |
|  | NSS-CT Item 04: Social Disruptions | **+++** | **0.546** | As expected |  |
|  | NSS-CT Item 08: Weekly Generalized Cataplexy Rate | + | 0.374 | Larger |  |
|  | NSS-CT Item 10: Work/Social Affected by Cataplexy | ++ | **0.576** | Larger |  |
|  | EQ-5D-5L Usual Activities | ++ | 0.444 | As expected |  |
|  | BC-CCI-E Total Score | ++ | 0.439 | As expected |  |
|  | BC-CCI-E Item 07: Job Difficulty | ++ | 0.459 | As expected |  |
|  | BC-CCI-E Item 08: Relationship Difficulty | ++ | **0.563** | Larger |  |
|  | BC-CCI-E Item 09: Social Difficulty | ++ | **0.638** | Larger |  |
|  | Weekly Cataplexy Rate | + | 0.384 | Larger |  |
|  | ESS Total Score | ++ | 0.275 | Smaller |  |
| **Everyday Activities** | SF-36 Role Physical | **+++** | -0.376 | Smaller |  |
|  | SF-36 Social Functioning | ++ | -0.294 | Smaller |  |
|  | SF-36 Vitality | ++ | -0.320 | As expected |  |
|  | NSS-CT Total Score | ++ | **0.522** | Larger |  |
|  | NSS-CT Item 03: Work Disruptions | ++ | 0.413 | As expected |  |
|  | NSS-CT Item 04: Social Disruptions | ++ | 0.442 | As expected |  |
|  | NSS-CT Item 08: Weekly Generalized Cataplexy Rate | + | 0.199 | As expected |  |
|  | NSS-CT Item 10: Work/Social Affected by Cataplexy | ++ | 0.380 | As expected |  |
|  | EQ-5D-5L Usual Activities | **+++** | 0.320 | Smaller |  |
|  | BC-CCI-E Total Score | ++ | 0.407 | As expected |  |
|  | BC-CCI-E Item 07: Job Difficulty | ++ | 0.327 | As expected |  |
|  | BC-CCI-E Item 08: Relationship Difficulty | ++ | 0.356 | As expected |  |
|  | BC-CCI-E Item 09: Social Difficulty | ++ | 0.429 | As expected |  |
|  | Weekly Cataplexy Rate | + | 0.253 | As expected |  |
|  | ESS Total Score | ++ | 0.493 | As expected |  |
| **Everyday Responsibilities** | SF-36 Role Physical | **+++** | **-0.520** | As expected |  |
|  | SF-36 Social Functioning | ++ | -0.436 | As expected |  |
|  | SF-36 Vitality | ++ | -0.440 | As expected |  |
|  | NSS-CT Total Score | ++ | 0.486 | As expected |  |
|  | NSS-CT Item 03: Work Disruptions | **+++** | **0.583** | As expected |  |
|  | NSS-CT Item 04: Social Disruptions | ++ | **0.548** | Larger |  |
|  | NSS-CT Item 08: Weekly Generalized Cataplexy Rate | + | 0.195 | As expected |  |
|  | NSS-CT Item 10: Work/Social Affected by Cataplexy | ++ | 0.410 | As expected |  |
|  | EQ-5D-5L Usual Activities | **+++** | **0.521** | As expected |  |
|  | BC-CCI-E Total Score | ++ | 0.470 | As expected |  |
|  | BC-CCI-E Item 07: Job Difficulty | ++ | **0.603** | Larger |  |
|  | BC-CCI-E Item 08: Relationship Difficulty | ++ | 0.445 | As expected |  |
|  | BC-CCI-E Item 09: Social Difficulty | ++ | 0.432 | As expected |  |
|  | Weekly Cataplexy Rate | + | 0.226 | As expected |  |
|  | ESS Total Score | ++ | 0.312 | As expected |  |

BC-CCI-E, British Columbia - Cognitive Complaints Inventory - Extended; EQ-5D-5L, EuroQol-5 Dimension 5-Level; ESS, Epworth Sleepiness Scale; FINI, Functional Impacts of Narcolepsy Instrument; NSS-CT, Narcolepsy Sleepiness Scale for Clinical Trials; SF-36, Short Form-36 Health Survey; WCR, weekly cataplexy rate.

For expected and observed correlations: + = small correlation (<0.3); ++ = low moderate correlation (0.3 – <0.5); +++ = high moderate to strong correlation (0.5+). Expected high moderate to strong correlations are indicated by gray shading. Observed high moderate to strong correlations are indicated in **bold**. Absolute values of the observed correlations larger or smaller than expected are indicated.

**Supplementary Table 4**. Responsiveness of the FINI domains relative to change in reference measures from baseline to Week 8 and Week 12

|  | | FINI Domain | | Reference Measure | | Change Correla-tion^a^ | | Change Level | | n | | Mean Change (SD) | | 95% CI | Within-Group SRM^b,c^ | Between-Group SRM^b,c^ |  |
| --- | --- | --- | --- | --- | --- | --- | --- | --- | --- | --- | --- | --- | --- | --- | --- | --- | --- |
|  | | **Tiredness** | | PGI-S | | Week 8  r = 0.647 | | Markedly improved | | 36 | | -59.1 (20.5) | | -66.1, -52.2 | -2.882*** |  |  |
|  | |  |  |  |  |  |  | Moderately improved | | 92 | | -49.0 (22.0) | | -53.5, -44.4 | -2.228*** | -0.472* |  |
|  | |  |  |  |  |  |  | Minimally improved | | 79 | | -27.4 (22.1) | | -32.3, -22.4 | -1.238*** | -0.981*** |  |
|  | |  |  |  |  |  |  | No change/worsened | | 56 | | -7.8 (25.3) | | -14.6, -1.1 | -0.310* | -0.831*** |  |
|  | |  |  |  |  | Week 12  r = 0.673 | | Markedly improved | | 36 | | -60.9 (18.0) | | -67.0, -54.8 | -3.390*** |  |  |
|  | |  |  |  |  |  |  | Moderately improved | | 73 | | -48.5 (23.2) | | -53.9, -43.1 | -2.093*** | -0.575** |  |
|  | |  |  |  |  |  |  | Minimally improved | | 84 | | -29.5 (21.9) | | -34.3, -24.8 | -1.348*** | -0.844*** |  |
|  | |  |  |  |  |  |  | No change/worsened | | 65 | | -8.1 (25.6) | | -14.5, -1.8 | -0.318* | -0.907*** |  |
|  | |  |  | SF-36 Vitality Item 03: Worn Out | | Week 8  r = -0.601 | | Markedly/moderately improved | | 79 | | -56.9 (22.2) | | -61.9, -51.9 | -2.561*** |  |  |
|  | |  |  |  |  |  |  | Minimally improved | | 68 | | -38.0 (22.4) | | -43.4, -32.6 | -1.700*** | -0.846*** |  |
|  | |  |  |  |  |  |  | No change/worsened | | 116 | | -18.6 (25.5) | | -23.3, -13.9 | -0.728*** | -0.797*** |  |
|  | |  |  |  |  | Week 12  r = -0.613 | | Markedly/moderately improved | | 80 | | -55.8 (20.7) | | -60.4, -51.1 | -2.689*** |  |  |
|  | |  |  |  |  |  |  | Minimally improved | | 65 | | -36.1 (20.7) | | -41.2, -31.0 | -1.742*** | -0.948*** |  |
|  | |  |  |  |  |  |  | No change/worsened | | 113 | | -17.1 (27.8) | | -22.3, -11.9 | -0.616*** | -0.747*** |  |
|  | |  |  | SF-36 Vitality Item 04: Tired | | Week 8  r = -0.754 | | Markedly/moderately improved | | 99 | | -58.3 (18.2) | | -61.9, -54.7 | -3.204*** |  |  |
|  | |  |  |  |  |  |  | Minimally improved | | 69 | | -36.2 (22.2) | | -41.6, -30.9 | -1.633*** | -1.107*** |  |
|  | |  |  |  |  |  |  | No change/worsened | | 95 | | -10.1 (20.1) | | -14.2, -6.0 | -0.503*** | -1.243*** |  |
|  | |  |  |  |  | Week 12  r = -0.760 | | Markedly/moderately improved | | 83 | | -59.1 (20.0) | | -63.4, -54.7 | -2.966*** |  |  |
|  | |  |  |  |  |  |  | Minimally improved | | 84 | | -34.9 (20.6) | | -39.3, -30.4 | -1.697*** | -1.197*** |  |
|  | |  |  |  |  |  |  | No change/worsened | | 91 | | -10.0 (22.6) | | -14.7, -5.3 | -0.442*** | -1.152*** |  |
| Cognitive Function-ing | | | PGI-S | | Week 8  r = 0.524 | | Markedly improved | | 36 | | -46.3 (28.8) | | -56.0, -36.5 | | -1.606*** |  | |
|  |  |  |  |  |  |  | Moderately improved | | 92 | | -37.6 (22.5) | | -42.2, -32.9 | | -1.668*** | -0.356 | |
|  |  |  |  |  |  |  | Minimally improved | | 79 | | -18.7 (23.7) | | -24.0, -13.4 | | -0.791*** | -0.816*** | |
|  |  |  |  |  |  |  | No change/worsened | | 56 | | -4.0 (24.3) | | -10.5, 2.5 | | -0.166 | -0.615*** | |
|  |  |  |  |  | Week 12  r = 0.582 | | Markedly improved | | 36 | | -48.1 (24.3) | | -56.3, -39.8 | | -1.980*** |  | |
|  |  |  |  |  |  |  | Moderately improved | | 73 | | -36.4 (25.9) | | -42.4, -30.3 | | -1.405*** | -0.461* | |
|  |  |  |  |  |  |  | Minimally improved | | 84 | | -21.8 (21.5) | | -26.5, -17.1 | | -1.012*** | -0.617*** | |
|  |  |  |  |  |  |  | No change/worsened | | 65 | | -4.0 (25.6) | | -10.3, 2.3 | | -0.156 | -0.761*** | |
|  |  |  | BC-CCI-E Item 01: Forgetful-ness/ Memory Problems | | Week 8  r = 0.624 | | Markedly/moderately improved | | 29 | | -64.3 (16.7) | | -70.7, -58.0 | | -3.844*** |  | |
|  |  |  |  |  |  |  | Minimally improved | | 93 | | -34.3 (24.0) | | -39.2, -29.3 | | -1.426*** | -1.334*** | |
|  |  |  |  |  |  |  | No change/worsened | | 141 | | -12.6 (23.1) | | -16.4, -8.8 | | -0.546*** | -0.924*** | |
|  |  |  |  |  | Week 12  r = 0.655 | | Markedly/moderately improved | | 26 | | -64.0 (22.6) | | -73.2, -54.9 | | -2.830*** |  | |
|  |  |  |  |  |  |  | Minimally improved | | 86 | | -33.6 (19.3) | | -37.7, -29.4 | | -1.743*** | -1.520*** | |
|  |  |  |  |  |  |  | No change/worsened | | 146 | | -13.2 (25.8) | | -17.4, -9.0 | | -0.511*** | -0.863*** | |
|  |  |  | BC-CCI-E Item 02: Poor Concentra-tion | | Week 8  r = 0.700 | | Markedly/moderately improved | | 63 | | -52.3 (24.9) | | -58.6, -46.0 | | -2.105*** |  | |
|  |  |  |  |  |  |  | Minimally improved | | 104 | | -30.1 (20.7) | | -34.1, -26.0 | | -1.453*** | -0.996*** | |
|  |  |  |  |  |  |  | No change/worsened | | 96 | | -4.2 (19.6) | | -8.2, -0.3 | | -0.215* | -1.281*** | |
|  |  |  |  |  | Week 12  r = 0.783 | | Markedly/moderately improved | | 53 | | -53.1 (22.8) | | -59.4, -46.8 | | -2.326*** |  | |
|  |  |  |  |  |  |  | Minimally improved | | 103 | | -32.8 (19.0) | | -36.5, -29.1 | | -1.727*** | -0.999*** | |
|  |  |  |  |  |  |  | No change/worsened | | 102 | | -2.8 (21.1) | | -6.9, 1.4 | | -0.132 | -1.494*** | |
|  |  |  | BC-CCI-E Item 03: Trouble Expressing Thoughts | | Week 8  r = 0.597 | | Markedly/moderately improved | | 40 | | -58.1 (22.9) | | -65.5, -50.8 | | -2.539*** |  | |
|  |  |  |  |  |  |  | Minimally improved | | 87 | | -30.4 (24.6) | | -35.7, -25.2 | | -1.234*** | -1.150*** | |
|  |  |  |  |  |  |  | No change/worsened | | 136 | | -13.6 (23.3) | | -17.6 -9.7 | | -0.586*** | -0.704*** | |
|  |  |  |  |  | Week 12  r = 0.586 | | Markedly/moderately improved | | 37 | | -55.1 (25.7) | | -63.7, -46.6 | | -2.148*** |  | |
|  |  |  |  |  |  |  | Minimally improved | | 80 | | -31.8 (21.0) | | -36.5, -27.1 | | -1.517*** | -1.034*** | |
|  |  |  |  |  |  |  | No change/worsened | | 141 | | -13.4 (25.7) | | -17.7, -9.1 | | -0.522*** | -0.764*** | |
|  |  |  | BC-CCI-E Item 05: Slow Thinking | | Week 8  r = 0.602 | | Markedly/moderately improved | | 52 | | -52.3 (24.7) | | -59.2, -45.4 | | -2.119*** |  | |
|  |  |  |  |  |  |  | Minimally improved | | 93 | | -31.0 (21.8) | | -35.5, -26.5 | | -1.421*** | -0.930*** | |
|  |  |  |  |  |  |  | No change/worsened | | 118 | | -10.3 (24.0) | | -14.7, -6.0 | | -0.431*** | -0.897*** | |
|  |  |  |  |  | Week 12  r = 0.711 | | Markedly/moderately improved | | 45 | | -56.2 (21.7) | | -62.8, -49.7 | | -2.588*** |  | |
|  |  |  |  |  |  |  | Minimally improved | | 94 | | -31.7 (21.2) | | -36.1, -27.4 | | -1.494*** | -1.147*** | |
|  |  |  |  |  |  |  | No change/worsened | | 119 | | -8.1 (23.0) | | -12.3, -3.9 | | -0.352*** | -1.061*** | |
| **Cata-plexy** | | | PGI-S | | Week 8  r = 0.525 | | Markedly improved | | 36 | | -34.0 (21.9) | | -41.5, -26.6 | | -1.551*** |  | |
|  |  |  |  |  |  |  | Moderately improved | | 92 | | -27.7 (22.4) | | -32.3, -23.0 | | -1.237*** | -0.286 | |
|  |  |  |  |  |  |  | Minimally improved | | 79 | | -13.3 (16.8) | | -17.1, -9.5 | | -0.790*** | -0.719*** | |
|  |  |  |  |  |  |  | No change/worsened | | 56 | | -0.5 (18.9) | | -5.6, 4.5 | | -0.028 | -0.720*** | |
|  |  |  |  |  | Week 12  r = 0.519 | | Markedly improved | | 36 | | -36.7 (20.0) | | -43.4, -29.9 | | -1.837*** |  | |
|  |  |  |  |  |  |  | Moderately improved | | 73 | | -29.5 (23.1) | | -34.9, -24.1 | | -1.274*** | -0.326 | |
|  |  |  |  |  |  |  | Minimally improved | | 84 | | -14.4 (19.0) | | -18.5, -10.2 | | -0.753*** | -0.718*** | |
|  |  |  |  |  |  |  | No change/worsened | | 65 | | -4.9 (17.9) | | -9.3, -0.4 | | -0.271* | -0.512** | |
|  |  |  | NSS-CT Item 10: Work/ Social Affected by Cataplexy | | Week 8  r = 0.611 | | Markedly/moderately improved | | 68 | | -35.7 (19.3) | | -40.4, -31.1 | | -1.853*** |  | |
|  |  |  |  |  |  |  | Minimally improved | | 103 | | -20.2 (20.6) | | -24.2, -16.1 | | -0.980*** | -0.777*** | |
|  |  |  |  |  |  |  | No change/worsened | | 92 | | -3.8 (18.8) | | -7.7, 0.2 | | -0.199 | -0.830*** | |
|  |  |  |  |  | Week 12  r = 0.590 | | Markedly/moderately improved | | 65 | | -36.1 (21.6) | | -41.4, -30.7 | | -1.674*** |  | |
|  |  |  |  |  |  |  | Minimally improved | | 101 | | -23.0 (18.3) | | -26.6, -19.4 | | -1.254*** | -0.667*** | |
|  |  |  |  |  |  |  | No change/worsened | | 92 | | -3.5 (18.5) | | -7.4, 0.3 | | -0.191 | -1.055*** | |
|  | **Social Activi-ties** | | PGI-S | | Week 8  r = 0.474 | | Markedly improved | | 36 | | -45.7 (26.0) | | -54.5, -36.9 | | -1.753*** |  | |
|  |  |  |  |  |  |  | Moderately improved | | 92 | | -39.1 (22.8) | | -43.8, -34.4 | | -1.716*** | -0.278 | |
|  |  |  |  |  |  |  | Minimally improved | | 79 | | -19.5 (25.3) | | -25.2, -13.9 | | -0.772*** | -0.814*** | |
|  |  |  |  |  |  |  | No change/worsened | | 56 | | -8.8 (28.6) | | -16.5, -1.2 | | -0.308* | -0.401* | |
|  |  |  |  |  | Week 12  r = 0.481 | | Markedly improved | | 36 | | -43.9 (23.8) | | -52.0, -35.9 | | -1.847*** |  | |
|  |  |  |  |  |  |  | Moderately improved | | 73 | | -38.7 (27.6) | | -45.1, -32.3 | | -1.404*** | -0.198 | |
|  |  |  |  |  |  |  | Minimally improved | | 84 | | -19.4 (22.7) | | -24.3, -14.5 | | -0.857*** | -0.769*** | |
|  |  |  |  |  |  |  | No change/worsened | | 65 | | -7.8 (30.8) | | -15.4, -0.2 | | -0.253* | -0.439** | |
|  |  |  | SF-36 Social Functioning Item 02: Physical/ Emotional Problems Interfered with Social Activities | | Week 8  r = -0.472 | | Markedly/moderately improved | | 80 | | -43.4 (26.3) | | -49.2, -37.5 | | -1.649*** |  | |
|  |  |  |  |  |  |  | Minimally improved | | 80 | | -29.1 (24.2) | | -34.5, -23.8 | | -1.205*** | -0.563*** | |
|  |  |  |  |  |  |  | No change/worsened | | 103 | | -14.3 (27.2) | | -19.6, -9.0 | | -0.526*** | -0.572*** | |
|  |  |  |  |  | Week 12  r = -0.475 | | Markedly/moderately improved | | 79 | | -45.0 (29.5) | | -51.6, -38.4 | | -1.525*** |  | |
|  |  |  |  |  |  |  | Minimally improved | | 66 | | -21.8 (23.1) | | -27.5, -16.1 | | -0.943*** | -0.867*** | |
|  |  |  |  |  |  |  | No change/worsened | | 113 | | -13.7 (26.2) | | -18.6, -8.8 | | -0.524*** | -0.321 | |
|  |  |  | NSS-CT Item 04: Social Disruptions by Sleep Attacks | | Week 8  r = 0.591 | | Markedly/moderately improved | | 101 | | -43.1 (23.1) | | -47.7, -38.6 | | -1.868*** |  | |
|  |  |  |  |  |  |  | Minimally improved | | 81 | | -30.5 (25.1) | | -36.0, -24.9 | | -1.215*** | -0.527*** | |
|  |  |  |  |  |  |  | No change/worsened | | 81 | | -5.6 (24.1) | | -10.9, -0.2 | | -0.230* | -1.013*** | |
|  |  |  |  |  | Week 12  r = 0.603 | | Markedly/moderately improved | | 91 | | -44.8 (25.5) | | -50.1, -39.5 | | -1.757*** |  | |
|  |  |  |  |  |  |  | Minimally improved | | 83 | | -25.8 (24.6) | | -31.1, -20.4 | | -1.046*** | -0.759*** | |
|  |  |  |  |  |  |  | No change/worsened | | 84 | | -3.9 (23.3) | | -9.0, 1.1 | | -0.169 | -0.910*** | |
|  | **Every-day Activi-ties** | | PGI-S | | Week 8  r = 0.621 | | Markedly improved | | 36 | | -59.4 (25.4) | | -68.0, -50.8 | | -2.336*** |  | |
|  |  |  |  |  |  |  | Moderately improved | | 92 | | -50.3 (19.8) | | -54.4, -46.2 | | -2.546*** | -0.420* | |
|  |  |  |  |  |  |  | Minimally improved | | 79 | | -30.5 (22.2) | | -35.5, -25.6 | | -1.373*** | -0.945*** | |
|  |  |  |  |  |  |  | No change/worsened | | 56 | | -10.8 (24.0) | | -17.2, -4.4 | | -0.452** | -0.858*** | |
|  |  |  |  |  | Week 12  r = 0.618 | | Markedly improved | | 36 | | -59.2 (25.2) | | -67.7, -50.7 | | -2.353*** |  | |
|  |  |  |  |  |  |  | Moderately improved | | 73 | | -48.3 (21.7) | | -53.3, -43.2 | | -2.230*** | -0.477* | |
|  |  |  |  |  |  |  | Minimally improved | | 84 | | -31.9 (20.1) | | -36.3, -27.6 | | -1.590*** | -0.786*** | |
|  |  |  |  |  |  |  | No change/worsened | | 65 | | -11.6 (28.1) | | -18.6, -4.7 | | -0.414** | -0.849*** | |
|  |  |  | EQ-5D-5L Usual Activities | | Week 8  r = 0.374 | | Markedly/moderately improved | | 71 | | -49.6 (24.7) | | -55.4, -43.7 | | -2.003*** |  | |
|  |  |  |  |  |  |  | Minimally improved | | 67 | | -40.1 (27.2) | | -46.7, -33.5 | | -1.477*** | -0.364* | |
|  |  |  |  |  |  |  | No change/worsened | | 125 | | -28.7 (27.3) | | -33.5, -23.8 | | -1.050*** | -0.421** | |
|  |  |  |  |  | Week 12  r = 0.483 | | Markedly/moderately improved | | 65 | | -52.2 (21.9) | | -57.6, -46.8 | | -2.386*** |  | |
|  |  |  |  |  |  |  | Minimally improved | | 73 | | -36.5 (27.6) | | -42.9, -30.0 | | -1.323*** | -0.628*** | |
|  |  |  |  |  |  |  | No change/worsened | | 120 | | -25.3 (28.1) | | -30.4, -20.2 | | -0.900*** | -0.400** | |
|  |  |  | SF-36 Role-Physical Item 04: Difficulty Performing Work | | Week 8  r = -0.458 | | Markedly/moderately improved | | 99 | | -51.4 (26.1) | | -56.6, -46.2 | | -1.968*** |  | |
|  |  |  |  |  |  |  | Minimally improved | | 64 | | -34.0 (22.5) | | -39.6, -28.4 | | -1.511*** | -0.703*** | |
|  |  |  |  |  |  |  | No change/worsened | | 100 | | -25.3 (26.8) | | -30.6, -19.9 | | -0.944*** | -0.347* | |
|  |  |  |  |  | Week 12  r = -0.501 | | Markedly/moderately improved | | 98 | | -49.8 (24.8) | | -54.8, -44.8 | | -2.009*** |  | |
|  |  |  |  |  |  |  | Minimally improved | | 67 | | -32.9 (24.4) | | -38.9, -27.0 | | -1.351*** | -0.686*** | |
|  |  |  |  |  |  |  | No change/worsened | | 93 | | -21.6 (28.1) | | -27.4, -15.8 | | -0.767*** | -0.427** | |
|  | **Every-day Respons-ibilities** | | PGI-S | | Week 8  r = 0.528 | | Markedly improved | | 36 | | -53.2 (30.1) | | -63.4, -43.1 | | -1.769*** |  | |
|  |  |  |  |  |  |  | Moderately improved | | 92 | | -46.2 (24.3) | | -51.2, -41.2 | | -1.905*** | -0.271 | |
|  |  |  |  |  |  |  | Minimally improved | | 79 | | -23.5 (25.8) | | -29.3, -17.7 | | -0.911*** | -0.907*** | |
|  |  |  |  |  |  |  | No change/worsened | | 56 | | -8.6 (29.0) | | -16.4, -0.9 | | -0.297* | -0.547** | |
|  |  |  |  |  | Week 12  r = 0.554 | | Markedly improved | | 36 | | -53.0 (28.5) | | -62.7, -43.4 | | -1.858*** |  | |
|  |  |  |  |  |  |  | Moderately improved | | 73 | | -46.5 (26.7) | | -52.7, -40.2 | | -1.742*** | -0.240 | |
|  |  |  |  |  |  |  | Minimally improved | | 84 | | -26.3 (24.5) | | -31.6, -21.0 | | -1.072*** | -0.790*** | |
|  |  |  |  |  |  |  | No change/worsened | | 65 | | -8.6 (31.0) | | -16.3, -0.9 | | -0.277* | -0.643*** | |
|  |  |  | EQ-5D-5L Usual Activities | | Week 8  r = 0.472 | | Markedly/moderately improved | | 71 | | -50.9 (27.4) | | -57.4, -44.4 | | -1.856*** |  | |
|  |  |  |  |  |  |  | Minimally improved | | 67 | | -38.1 (26.3) | | -44.5, -31.7 | | -1.448*** | -0.479** | |
|  |  |  |  |  |  |  | No change/worsened | | 125 | | -18.7 (29.3) | | -23.9, -13.6 | | -0.639*** | -0.683*** | |
|  |  |  |  |  | Week 12  r = 0.484 | | Markedly/moderately improved | | 65 | | -49.7 (28.6) | | -56.8, -42.7 | | -1.739*** |  | |
|  |  |  |  |  |  |  | Minimally improved | | 73 | | -33.1 (27.3) | | -39.5, -26.8 | | -1.215*** | -0.596** | |
|  |  |  |  |  |  |  | No change/worsened | | 120 | | -20.1 (31.4) | | -25.8, -14.5 | | -0.641*** | -0.434** | |
|  |  |  | SF-36 Role-Physical Item 04: Difficulty Performing Work | | Week 8  r = -0.521 | | Markedly/moderately improved | | 99 | | -52.5 (26.8) | | -57.9, -47.2 | | -1.961*** |  | |
|  |  |  |  |  |  |  | Minimally improved | | 64 | | -22.7 (21.1) | | -27.9, -17.4 | | -1.075*** | -1.209*** | |
|  |  |  |  |  |  |  | No change/worsened | | 100 | | -18.6 (30.3) | | -24.6, -12.6 | | -0.613*** | -0.150 | |
|  |  |  |  |  | Week 12  r = -0.541 | | Markedly/moderately improved | | 98 | | -51.1 (25.9) | | -56.3, -45.9 | | -1.975*** |  | |
|  |  |  |  |  |  |  | Minimally improved | | 67 | | -22.4 (24.3) | | -28.3, -16.5 | | -0.920*** | -1.137*** | |
|  |  |  |  |  |  |  | No change/worsened | | 93 | | -16.8 (31.8) | | -23.3, -10.2 | | -0.527*** | -0.195 | |
|  |  |  | NSS-CT Item 03: Work Disruptions by Sleep Attacks | | Week 8  r = 0.625 | | Markedly/moderately improved | | 120 | | -50.1 (24.0) | | -54.4, -45.7 | | -2.088*** |  | |
|  |  |  |  |  |  |  | Minimally improved | | 71 | | -27.4 (24.7) | | -33.2, -21.5 | | -1.106*** | -0.937*** | |
|  |  |  |  |  |  |  | No change/worsened | | 72 | | -7.8 (29.2) | | -14.6, -0.9 | | -0.266* | -0.724*** | |
|  |  |  |  |  | Week 12  r = 0.602 | | Markedly/moderately improved | | 116 | | -49.6 (23.8) | | -54.0, -45.2 | | -2.081*** |  | |
|  |  |  |  |  |  |  | Minimally improved | | 73 | | -24.9 (29.0) | | -31.6, -18.1 | | -0.860*** | -0.952*** | |
|  |  |  |  |  |  |  | No change/worsened | | 69 | | -7.3 (28.0) | | -14.0, -0.5 | | -0.259* | -0.619*** | |

|  | *P*<0.05 (*). |
| --- | --- |
|  | *P*<0.01 (**). |
|  | *P*<0.01 (***). |

ANOVA, analysis of variance; BC-CCI-E, British Columbia Cognitive Complaints Inventory - Extended; CGI-S, Clinician Global Impression of Severity; EQ-5D-5L, EuroQol-5 Dimension 5-Level; FINI, Functional Impacts of Narcolepsy Instrument; NSS-CT, Narcolepsy Sleepiness Scale for Clinical Trials; PGI-S, Patient Global Impression of Severity; SD, standard deviation; SRM, standardized response mean.

^a^Correlations provided are between change in FINI domain scores and change in reference measures from baseline to Week 8 and Week 12.

^b^For within groups, SRM is calculated by dividing the mean change score between baseline and the subsequent time point by the SD of the change score. For between groups, the SRM is calculated by dividing the difference in change scores between consecutive groups by the pooled change SD. SRM values: small change = 0.2, moderate change = 0.5, large change = 0.8.

^c^The *P*-value for each individual change group is derived from a paired t-test assessing the difference in the same group over time (for “within-group” comparisons). The *P*-value for the comparisons to the prior (less improved) adjacent severity group is derived from select pairwise comparisons (independent samples t-test) under ANOVA to assess difference in score change between groups (for “between-group” comparisons). *P*-values: **P*<0.05, ***P*<0.01, ****P*<0.001.

**Supplementary Table 5**. Correlations between FINI domain scores and prespecified anchor measures for change from baseline at Week 12

**TAK-861-3001 study**

|  |  | FINI Domain | | | | | |
| --- | --- | --- | --- | --- | --- | --- | --- |
| **Anchor Measure** | **N** | **Tiredness** | **Cognitive Functioning** | **Cataplexy** | **Social Activities** | **Everyday Activities** | **Everyday Responsibil-ities** |
| **Global Impression Anchors** | | | | | | | |
| PGI-S | 157 | 0.674 | 0.580 | 0.483 | 0.452 | 0.554 | 0.487 |
| CGI-S | 154 | 0.529 | 0.439 | 0.429 | 0.484 | 0.494 | 0.422 |
| PGI-C | 157 | 0.707 | 0.588 | 0.437 | 0.440 | 0.663 | 0.470 |
| CGI-C | 156 | 0.627 | 0.591 | 0.455 | 0.508 | 0.626 | 0.507 |
| **Concept-Specific Anchors** | | | | | | | |
| EQ-5D-5L Usual Activities | 157 | - | - | - | - | 0.450 | 0.499 |
| NSS-CT Item 03: Work Disruptions | 157 | - | - | - | - | - | 0.550 |
| NSS-CT Item 04: Social Disruptions | 157 | - | - | - | 0.581 |  | - |
| NSS-CT Item 10: Work/Social Affected by Cataplexy | 157 | - | - | 0.540 | - | - | - |
| SF-36 Social Functioning Item 02: Physical/Emotional Problems Interfered with Social Activities | 157 | - | - | - | -0.428 | - | - |
| SF-36 Role-Physical Item 04: Difficulty Performing Work | 157 | - | - | - | - | -0.511 | -0.494 |
| SF-36 Vitality Item 03: Worn Out | 157 | -0.608 | - | - | - | - | - |
| SF-36 Vitality Item 04: Tired | 157 | -0.750 | - | - | - | - | - |
| BC-CCI-E Item 01: Forgetfulness/Memory Problems | 157 | - | 0.581 | - | - | - | - |
| BC-CCI-E Item 02: Poor Concentration | 157 | - | 0.773 | - | - | - | - |
| BC-CCI-E Item 03: Trouble Expressing Thoughts | 157 | - | 0.579 | - | - | - | - |
| BC-CCI-E Item 05: Slow Thinking | 157 | - | 0.704 | - | - | - | - |
| Percent Change in WCR | 157 | - | - | 0.386 | - | - | - |

**TAK-861-3002 Study**

|  |  | FINI Domain | | | | | |
| --- | --- | --- | --- | --- | --- | --- | --- |
| **Anchor Measure** | **N** | **Tiredness** | **Cognitive Functioning** | **Cataplexy** | **Social Activities** | **Everyday Activities** | **Everyday Responsibil-ities** |
| **Global Impression Anchors** | | | | | | | |
| PGI-S | 101 | 0.673 | 0.585 | 0.572 | 0.527 | 0.703 | 0.647 |
| CGI-S | 100 | 0.681 | 0.494 | 0.389 | 0.458 | 0.645 | 0.623 |
| PGI-C | 101 | 0.789 | 0.656 | 0.577 | 0.657 | 0.784 | 0.709 |
| CGI-C | 100 | 0.612 | 0.436 | 0.314 | 0.416 | 0.620 | 0.560 |
| **Concept-Specific Anchors** | | | | | | | |
| EQ-5D-5L Usual Activities | 101 | - | - | - | - | 0.520 | 0.448 |
| NSS-CT Item 03: Work Disruptions | 101 | - | - | - | - | - | 0.666 |
| NSS-CT Item 04: Social Disruptions | 101 | - |  | - | 0.635 | - | - |
| NSS-CT Item 10: Work/Social Affected by Cataplexy | 101 | - | - | 0.663 | - | - | - |
| SF-36 Social Functioning Item 02: Physical/Emotional Problems Interfered with Social Activities | 101 | - | - | - | -0.556 | - | - |
| SF-36 Role-Physical Item 04: Difficulty Performing Work | 101 | - | - | - | - | -0.505 | -0.612 |
| SF-36 Vitality Item 03: Worn Out | 101 | -0.626 | - | - | - | - | - |
| SF-36 Vitality Item 04: Tired | 101 | -0.786 | - | - | - | - | - |
| BC-CCI-E Item 01: Forgetfulness/Memory Problems | 101 | - | 0.758 | - | - | - | - |
| BC-CCI-E Item 02: Poor Concentration | 101 | - | 0.803 | - | - | - | - |
| BC-CCI-E Item 03: Trouble Expressing Thoughts | 101 | - | 0.598 | - | - | - | - |
| BC-CCI-E Item 05: Slow Thinking | 101 | - | 0.716 | - | - | - | - |
| Percent Change in WCR | 98 | - | - | 0.525 | - | - | - |

BC-CCI-E, British Columbia Cognitive Complaints Inventory – Extended; CGI-C, Clinician Global Impression of Change; CGI-S, Clinician Global Impression of Severity; EQ-5D-5L, EuroQol 5-Dimension 5-Level; FINI, Functional Impacts of Narcolepsy Instrument; NSS-CT, Narcolepsy Severity Scale for Clinical Trials; PGI-C, Patient Global Impression of Change; PGI-S, Patient Global Impression of Severity; SF-36, Short Form-36 Health Survey; WCR, weekly cataplexy rate.

**Supplementary Table 6**. Collapsed within-group anchor-based meaningful change derivation for the FINI domains with PGI-S as an anchor for change from baseline at Week 12

|  | Study 3001 | | | | | Study 3002 | | | | |
| --- | --- | --- | --- | --- | --- | --- | --- | --- | --- | --- |
| **PGI-S Anchor category** | **Corr^a^** | **n** | **Mean Change (SD)** | **Median** | **95% CI** | **Corr** | **n** | **Mean Change (SD)** | **Median** | **95% CI** |
|  | **FINI Tiredness** | | | | | **FINI Tiredness** | | | | |
| Much improved: ≤-3 | 0.673 | 21 | -65.0 (16.4) | -64.3 | -72.4, -57.5 | 0.656 | 15 | -55.2 (19.1) | -53.6 | -65.8, -44.7 |
| Improved: -2 |  | 43 | -49.3 (23.6) | -50.0 | -56.5, -42.0 |  | 30 | -47.4 (22.9) | -50.0 | -55.9, -38.9 |
| Minimally improved: -1 |  | 55 | -30.4 (21.3) | -32.1 | -36.2, -24.6 |  | 29 | -27.8 (23.2) | -25.0 | -36.7, -19.0 |
| Stable/Worsened: ≥0 |  | 38 | -9.4 (26.5) | -7.1 | -18.1, -0.7 |  | 27 | -6.3 (24.7) | -7.1 | -16.1, 3.4 |
|  | | **FINI Cognitive Functioning** | | | | **FINI Cognitive Functioning** | | | | |
| Much improved: ≤-3 | 0.563 | 21 | -49.5 (25.5) | -45.0 | -61.1, -37.9 | 0.567 | 15 | -46.0 (23.2) | -45.0 | -58.8, -33.2 |
| Improved: -2 |  | 43 | -39.0 (27.0) | -35.0 | -47.3, -30.7 |  | 30 | -32.7 (24.2) | -35.0 | -41.7, -23.6 |
| Minimally improved: -1 |  | 55 | -21.3 (18.9) | -25.0 | -26.4, -16.2 |  | 29 | -22.8 (26.2) | -20.0 | -32.7, -12.8 |
| Stable/Worsened: ≥0 |  | 38 | -6.4 (26.0) | 0.0 | -15.0, 2.1 |  | 27 | -0.6 (25.0) | 0.0 | -10.5, 9.3 |
|  | **FINI Cataplexy** | | | | | **FINI Cataplexy** | | | | |
| Much improved: ≤-3 | 0.476 | 21 | -34.8 (19.4) | -35.0 | -43.6, -25.9 | 0.580 | 15 | -39.3 (21.1) | -40.0 | -51.0, -27.6 |
| Improved: -2 |  | 43 | -30.8 (24.7) | -30.0 | -38.4, -23.2 |  | 30 | -27.5 (21.0) | -30.0 | -35.3, -19.7 |
| Minimally improved: -1 |  | 55 | -13.2 (18.4) | -10.0 | -18.2, **-8.2** |  | 29 | -16.6 (20.3) | -15.0 | -24.3, -8.8 |
| Stable/Worsened: ≥0 |  | 38 | -6.8 (18.9) | -5.0 | **-13.0**, -0.6 |  | 27 | -2.0 (16.4) | -5.0 | -8.5, 4.4 |
|  | **FINI Social Activities** | | | | | **FINI Social Activities** | | | | |
| Much improved: ≤-3 | 0.440 | 21 | -47.6 (23.3) | -50.0 | -58.2, -37.0 | 0.535 | 15 | -38.8 (24.2) | -37.5 | -52.2, -25.3 |
| Improved: -2 |  | 43 | -37.9 (27.3) | -31.3 | -46.3, -29.5 |  | 30 | -39.8 (28.4) | -34.4 | -50.4, -29.2 |
| Minimally improved: -1 |  | 55 | -19.5 (19.9) | -12.5 | -24.9, **-14.2** |  | 29 | -19.2 (27.6) | -12.5 | -29.7, -8.7 |
| Stable/Worsened: ≥0 |  | 38 | -13.5 (35.5) | -6.3 | **-25.2**, -1.8 |  | 27 | 0.2 (20.5) | 0.0 | -7.9, 8.3 |
|  | | **FINI Everyday Activities** | | | | **FINI Everyday Activities** | | | | |
| Much improved: ≤-3 | 0.556 | 21 | -58.3 (28.9) | -68.8 | -71.5, -45.2 | 0.704 | 15 | -60.4 (19.7) | -68.8 | -71.3, -49.5 |
| Improved: -2 |  | 43 | -48.5 (20.8) | -50.0 | -54.9, -42.2 |  | 30 | -47.9 (23.2) | -50.0 | -56.6, -39.2 |
| Minimally improved: -1 |  | 55 | -32.0 (19.7) | -31.3 | -37.4, -26.7 |  | 29 | -31.7 (21.1) | -31.3 | -39.7, -23.7 |
| Stable/Worsened: ≥0 |  | 38 | -16.9 (29.3) | -12.5 | -26.6, -7.3 |  | 27 | -4.2 (24.9) | 0.0 | -14.0, 5.7 |
|  | | **FINI Everyday Responsibilities** | | | | **FINI Everyday Responsibilities** | | | | |
| Much improved: ≤-3 | 0.470 | 21 | -50.8 (30.0) | -50.0 | -64.5, -37.1 | 0.644 | 15 | -56.1 (27.0) | -50.0 | -71.1, -41.2 |
| Improved: -2 |  | 43 | -48.8 (26.8) | -50.0 | -57.1, -40.6 |  | 30 | -43.1 (26.5) | -45.8 | -53.0, -33.2 |
| Minimally improved: -1 |  | 55 | -27.7 (24.5) | -25.0 | -34.4, **-21.1** |  | 29 | -23.6 (24.7) | -16.7 | -33.0, -14.2 |
| Stable/Worsened: ≥0 |  | 38 | -14.5 (32.1) | -4.2 | **-25.0**, -3.9 |  | 27 | -0.3 (27.9) | 0.0 | -11.3, 10.7 |

CI, confidence interval; FINI, Functional Impacts of Narcolepsy Instrument; PGI-S, Patient Global Impression of Severity; SD, standard deviation.

CI values in **bold** indicate overlap between minimally improved and stable/worsened categories.

^a^The correlation is the polyserial correlation coefficient between the change from Baseline on the FINI domains and the change from Baseline in *collapsed* categories of the PGI-S anchor measure.

**Supplementary Table 7**. Summary of meaningful change (Minimal Improvement) for each FINI domain at Week 12

|  |  | | Study 3001 | | | | | | | Study 3002 | | | | | |
| --- | --- | --- | --- | --- | --- | --- | --- | --- | --- | --- | --- | --- | --- | --- | --- |
|  |  | | **Simple Mean** | **Weighted* Estimates** | | | | | | **Simple Mean** | | **Weighted* Estimates** | | | |
| **FINI Domain** | **Anchors** | |  | **Mean** | | **Median** | | | **95% CI** |  |  | **Mean** | **Median** | **95% CI** | |
| **Tiredness** | Global impression | | -25.6 | -25.5 | | -25.4 | | | -33.6, -17.4 | -34.0 | | -33.9 | -33.4 | -41.5, -26.3 | |
|  | Concept specific | | -35.2 | -35.1 | | -37.3 | | | -42.0, -28.3 | -35.9 | | -35.9 | -41.0 | -42.7, -29.0 | |
|  | Overall | | -28.8 | -28.8 | | -29.5 | | | -36.5, -21.1 | -34.7 | | -34.6 | -36.0 | -41.9, -27.2 | |
|  | **MWPC estimate (range)** | |  | **-35** | |  | | | **(-30 to -40)** |  | | **-35** |  | **(-30 to -40)** | |
| **Cognitive Functioning** | Global impression | | -18.9 | -19.0 | | -19.6 | | | -27.2, -10.8 | -25.7 | | -25.4 | -21.6 | -34.8, -15.8 | |
|  | Concept specific | | -32.6 | -32.8 | | -31.5 | | | -38.3, -27.3 | -30.5 | | -30.7 | -30.1 | -37.4, -23.9 | |
|  | Overall | | -25.7 | -26.4 | | -26.0 | | | -33.2, -19.7 | -28.1 | | -28.4 | -26.4 | -36.3, -20.4 | |
|  | **MWPC estimate (range)** | |  | **-30** | |  | | | **(-25 to -35)** |  | | **-30** |  | **(-25 to -35)** | |
|  | Global impression | -13.8 | | | **-13.8** | | -10.6 | -20.9, -6.7 | | -18.5 | -17.6 | | -18.4 | | -25.3, -9.7 |
| **Cataplexy** | Concept specific | | -19.6 | -20.2 | | -18.0 | | | -25.8, -14.6 | -22.9 | | -22.9 | -20.0 | -30.2, -15.7 | |
|  | Overall | | -15.7 | -15.9 | | -13.1 | | | -22.5, -9.3 | -19.9 | | -19.6 | -19.0 | -27.1, -12.0 | |
|  | **MWPC estimate (range)** | |  | **-20** | |  | | | **(-15 to -25)** |  | | **-20** |  | **(-15 to -25)** | |
| **Social Activities** | Global impression | | -17.7 | -17.6 | | -14.0 | | | -26.2, -9.1 | -25.0 | | -24.4 | -23.2 | -34.8, -13.9 | |
|  | Concept specific | | -23.8 | -23.9 | | -23.6 | | | -30.5, -17.3 | -23.4 | | -23.9 | -26.8 | -34.8, -13.0 | |
|  | Overall | | -19.8 | -19.8 | | -17.4 | | | -27.7, -12.0 | -24.4 | | -24.2 | -24.5 | -34.8, -13.6 | |
|  | **MWPC estimate (range)** | |  | **-25** | |  | | | **(-20 to -30)** |  | | **-25** |  | **(-20 to -35)** | |
| **Everyday Activities** | Global impression | | -30.6 | -30.6 | | -29.7 | | | -37.7, -23.4 | -35.4 | | -35.3 | -35.8 | -43.9, -26.8 | |
|  | Concept specific | | -32.0 | -31.7 | | -31.3 | | | -38.9, -24.5 | -40.1 | | -40.1 | -43.6 | -51.9, -28.3 | |
|  | Overall | | -31.0 | -30.9 | | -30.1 | | | -38.0, -23.7 | -36.9 | | -36.5 | -37.7 | -45.8, -27.1 | |
|  | **MWPC estimate (range)** | |  | **-35** | |  | | | **(-30 to -40)** |  | | **-35** |  | **(-30 to -40)** | |
| **Everyday Responsibilities** | Global impression | | -27.3 | -27.4 | | -30.3 | | | -38.5, -16.2 | -30.8 | | -30.6 | -31.2 | -39.8, -21.5 | |
|  | Concept specific | | -25.2 | -25.0 | | -30.5 | | | -32.8, -17.3 | -29.8 | | -27.7 | -26.7 | -39.5, -16.0 | |
|  | Overall | | -26.4 | -26.3 | | -30.4 | | | -36.0, -16.7 | -30.3 | | -29.5 | -29.5 | -39.7, -19.4 | |
|  | **MWPC estimate (range)** | |  | **-30** | |  | | | **(-25 to -35)** |  | | **-30** |  | **(-25 to -35)** | |

CI, confidence interval; FINI, Functional Impacts of Narcolepsy Instrument; MWPC, meaningful within-patient change.

*Weighted by change correlation with the anchor. The correlation is the polyserial correlation coefficient between the change from Baseline on the FINI domains and the change from Baseline in *collapsed* categories of each anchor measure.

**List of Institutional Review Boards and/or Independent Ethics Committees used by investigators in The First Light (TAK-861-3001) and The Radiant Light (TAK-861-3002)**

**The First Light**

CEIM Hospital Universitario Vall d’Hebron

Passeig de la Vall d.Hebron

119-129 Edificio Materno-Infantil

Planta 13

Barcelona, 08035

Spain

Comitato Etico Area Vasta Emilia Centro (AVEC)

IRCCS Azienda Ospedaliero–Universitaria di Bologna,

Policlinico S.Orsola-Malpighi,

Via Albertoni, 15

40138 Bologna

Italy

Comite de Protection des Personnes Ile de France VII

Hopital de Bicetre

78 Rue du General Leclerc

Le Kremlin Bicetre, 94270

France

Ethik-Kommission der Medizinischen Fakultat der Rheinischen

Friedrich-Wil-helms-Universitat Bonn

Gebaude 74/4. OG

Venusberg-Campus 1

Bonn, 53127

Germany

Ethikkommission Norwest- und Zentralschweiz EKNZ

Geschaftsfuhrerin Irene Oberli

Tellplatz 11, Basel, 4053

Switzerland

Health Research Authority

East Midlands-Leicester Central Research Ethics Committee

2 Redman Place

Stratford, London, E20 1JQ

United Kingdom

Review Board of Human Rights and Ethics for Clinical Studies

Institutional Review Board

2-2-1, Kyobashi

Chuo-ku, Tokyo

Japan

Stitching Beoordeling Ethiek Biomedisch Onderzoek

Weiersstraat 1C

9401 ET, Assen

The Netherlands

The Norwegian Ethics Committees for Clinical Trials on

Medicinal Products and Medical Devices

PO Box 1130

Blindern, 0318 Oslo

Norway

WCG IRB

212 Carnegie Center

Suite 301

Princeton, New Jersey, 08540

United States

Nord-de-l'Ile-de-Montreal CIUSSS REB

5400 boul. Gouin ouest, J-2050

Montreal, Quebec, H4J 1C5

Canada

Cleveland Clinic IRB

9500 Euclid Ave.

OS-1

Cleveland, Ohio, 44195

United States

Mayo Clinic Institutional Review Board

200 First Street SW

Rochester, Minnesota, 55905

United States

National Center of Neurology and Psychiatry Institutional

Review Board

4-1-1 Ogawa-higashi-cho

Kodaira-shi, Tokyo

Japan

Beth Israel Deaconess Medical Center Committee on Clinical

Investigations

330 Brookline Ave.

Boston, Massachusetts, 02215

United States

Research Compliance Office

Stanford University

1705 El Camino Real

Palo Alto, California, 94306

United States

Gokeikai Osaka Kaisei Hospital Institutional Review Board

1-6-10 Miyahara Yodogawa-ku

Osaka-shi, Osaka-fu

Japan

**The Radiant Light**

Naczelna Komisja Bioetyczna

00–801 Warszawa

ul. Chmielna 69

Poland

Hôpital Universitaire de Bruxelles (H.U.B)

Secrétariat du Comité d'Ethique

Ruel Meylemeersch 90

B-1070 Bruxelles

Belgium

Comité de Protection des Personnes Île de France VI

Hôpital de la Pitié Salpêtrière

47, boulevard de l'Hôpital

Paris Cedex 13 75651

France

CEIm Hospital Clinic de Barcelona

Villarroel, 170 - Planta 0 - Puerta 6b

Barcelona, 08036

Spain

Etikprövningsmyndigheten

Box 2110

750 02 Uppsala

Sweden

Ethikkommission der Medizinischen Universität Innsbruck

Geschäftsstelle der Ethikkommission der Medizinischen

Universität Innsbruck

Anichstraße 35

6020 Innsbruck

Austria

Comitato Etico Area Vasta Emilia Centro (AVEC)

IRCCS Azienda Ospedaliero – Universitaria di Bologna,

Policlinico S.Orsola-Malpighi,

Via Albertoni, 15

40138 Bologna

Italy

Tukija

Valvira

PL 43

00521 Helsinki

Finland

Keimyung University Dongsan Hospital IRB

1035 Dalgubeol-daero, Dalseo-gu

Dalseo District, Daegu, 42601

South Korea

The Catholic University of Korea, St. Vincent’s Hospital IRB

93 Jungbu-daero Paldal-gu

Paldal-gu, Suwon-si, Gyeonggi-do, 16247

South Korea

Seoul National University Hospital IRB

101 Daehak-ro

Jongno-gu, Seoul, 03080

South Korea

Bellberry Human Research Ethics Committee

123 Glen Osmond Road

Eastwood, SA, 5063

Australia

Ethics Review Committee of Huashan Hospital Fudan

University (HIRB)

12 Urumqi Middle Road

Shanghai, 200040

China

Medical Ethics Committee of NanFang Hospital of Southern

Medical University

1838 Guangzhou Blvd North

Baiyun, Guangzhou, Guangdong Province, 510515

China

Henan Provincial People’s Hospital Drug (Device) Clinical

Trial Ethics Committee

7 Weiwu Road

Jin Shui Qu, Zheng Zhou Shi, He Nan Sheng, 450003

China

Ethics Committee of Xuanwu Hospital, Capital Medical

University

45 Changchun Street

Xicheng District, Beijing, 100053

China
